# Application of Spatial Omics in the Cardiovascular System

**DOI:** 10.34133/research.0628

**Published:** 2025-03-08

**Authors:** Yuhong Hu, Hao Jia, Hao Cui, Jiangping Song

**Affiliations:** ^1^ Department of Cardiac Surgery, Fuwai Hospital, National Center for Cardiovascular Diseases, Chinese Academy of Medical Sciences and Peking Union Medical College, Beijing, China.; ^2^ State Key Laboratory of Cardiovascular Disease, Fuwai Hospital, National Center for Cardiovascular Diseases, Chinese Academy of Medical Sciences and Peking Union Medical College, Beijing, China.; ^3^ Beijing Key Laboratory of Preclinical Research and Evaluation for Cardiovascular Implant Materials, Fuwai Hospital, National Center for Cardiovascular Diseases, Chinese Academy of Medical Sciences and Peking Union Medical College, Beijing, China.; ^4^ Department of Cardiac Surgery, Fuwai Yunnan Hospital, Chinese Academy of Medical Sciences, Affiliated Cardiovascular Hospital of Kunming Medical University, Kunming, China.; ^5^ Shenzhen Key Laboratory of Cardiovascular Disease, Fuwai Hospital, Chinese Academy of Medical Sciences, Shenzhen, China.

## Abstract

Cardiovascular diseases constitute a marked threat to global health, and the emergence of spatial omics technologies has revolutionized cardiovascular research. This review explores the application of spatial omics, including spatial transcriptomics, spatial proteomics, spatial metabolomics, spatial genomics, and spatial epigenomics, providing more insight into the molecular and cellular foundations of cardiovascular disease and highlighting the critical contributions of spatial omics to cardiovascular science, and discusses future prospects, including technological advancements, integration of multi-omics, and clinical applications. These developments should contribute to the understanding of cardiovascular diseases and guide the progress of precision medicine, targeted therapies, and personalized treatments.

## Introduction

Cardiovascular diseases remain one of the leading causes of mortality globally, with incidence rates continuing to rise annually in most countries [1]. Consequently, the study of cardiovascular system diseases is a critical area of medical research. Recent advances in spatial omics technologies have revolutionized this field by providing an unprecedented view of the cellular and molecular mechanisms underlying heart disease. By integrating high-throughput omics with spatial resolution, these revolutionary approaches enable researchers to map the transcriptional, proteomic, and metabolic states of cells within the complex architecture of cardiovascular tissues [2]. Spatial omics comprises 5 main branches: spatial transcriptomics, spatial proteomics, spatial metabolomics, spatial genomics, and spatial epigenomics [3]. In addition, the genesis of spatial omics has enabled a multidimensional understanding of cardiovascular pathology. By capturing the heterogeneity and dynamic interplay of cells in situ, spatial omics technologies have unveiled novel cellular communities, metabolic landscapes, and molecular mechanisms underlying cardiovascular health and disease [4]. This review summarizes the contributions of spatial omics to cardiovascular science, emphasizing how these technologies elucidate the complex biological processes of the cardiovascular diseases and guide the development of novel therapeutic strategies.

## Overview of Spatial Omics Technologies

**Fig. 1.**
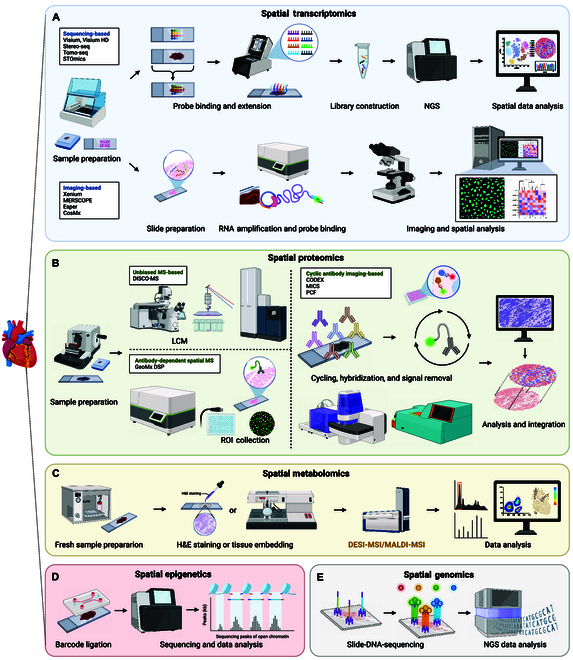
Schematic principle and workflow of spatial omics in cardiovascular research. (A) Spatial transcriptomics: Mapped gene expression patterns in cardiovascular tissues, identifying specific gene signatures in different heart regions. Sequencing-based methods are illustrated using the principles of Visium as an example, while imaging-based methods are represented by the principles of Xenium. Slight variations in principles may exist across different technologies. (B) Spatial proteomics: Analyzed protein distributions and interactions, revealing protein networks and spatial organization within cardiac tissues. Antibody-dependent spatial MS methods are illustrated using the principles of GeoMx DSP as an example, while cyclic antibody imaging-based methods are represented by the principles of PCF and CODEX. Slight variations in principles may exist across different technologies. (C) Spatial metabolomics: Provided high-resolution metabolite profiling, revealing metabolic alterations and spatial distributions in cardiovascular tissues. (D) Spatial epigenomics: Profiled chromatin accessibility, histone modifications, and DNA methylation patterns, providing insights into epigenetic landscapes in cardiac cells. (E) Spatial genomics: Studied the spatial arrangement of genetic material within cell nuclei, revealing the 3D genome organization and its impact on cellular function. Created with BioRender.

Spatial omics aims to elucidate how the precise localization and spatial relationships of cells within tissues influence their function and interactions. By integrating genomic, transcriptomic, and proteomic methodologies with high-resolution imaging and molecular labeling, researchers can reveal the molecular characteristics of cells in situ at the single-cell level. These technologies unveil the complex interplay and functional evolution among cells, offering new perspectives and potential modalities for disease diagnosis and treatment. The principles and workflow of the 5 spatial omics are shown in Fig. 1.

### Single-cell omics: Gaining cellular insight but losing spatial information

Single-cell RNA sequencing (scRNA-seq) has revolutionized molecular biology by enabling high-throughput profiling of gene expression at the resolution of individual cells. It has become a cornerstone for studying cellular heterogeneity, identifying rare cell populations, and understanding dynamic cellular states in development and disease [5]. The workflow involves isolating individual cells, capturing RNA, and sequencing the resulting cDNA, producing comprehensive transcriptomic profiles for each cell [6]. This method has been widely adopted in studies of complex tissues, such as the tumor microenvironment, the immune system, and organ development, providing insights into the molecular signatures of diverse cell populations. For example, Villani et al. [7] discovered new types of blood dendritic cells (DCs) with the ability to activate T cells.

Another alternative method is single-nucleus RNA sequencing (snRNA-seq), which involves extracting RNA from individual cell nuclei and is suitable for processing frozen, fixed, or difficult-to-dissociate tissue samples [8]. Due to the larger size of cardiomyocytes (CMs) compared to typical cells, they are unable to be effectively processed by droplet-based scRNA-seq platforms [9]. Consequently, snRNA-seq exhibits a higher capture efficiency for CMs than scRNA-seq. Litviňuková et al. [10] have utilized this technique to map the cellular atlas of the healthy human heart.

Despite its transformative impact, scRNA-seq and snRNA-seq have notable limitations. The dissociation of tissues during sample preparation disrupts the spatial context of cells, erasing critical information about their interactions and positional cues as described by Wang and Navin [11]. Additionally, tissue dissociation can introduce transcriptional artifacts and bias, and it is neccessary to remove cell debris and contaminition or rare cell types may be lost during the process. However, the requirement for removal of contamination dead cell to acquire high-quality sample for scRNA-seq can potentially stress the cells and alter the transcriptional profile [12]. These constraints make it challenging to study processes where spatial organization plays a key role, such as organogenesis or immune responses. Furthermore, scRNA-seq and snRNA-seq typically provide limited insight into the tissue architecture or the physical relationships between cells [13].

These limitations highlight the need for complementary technologies that retain spatial information while profiling gene expression. Spatial omics methods address this gap, enabling researchers to integrate molecular and spatial data for a more holistic understanding of biological systems.

### The explosive emergence of spatial omics methodologies in the past decade

Spatial omics is a transformative approach that integrates various omics techniques, transcriptomics, proteomics, metabolomics, epigenomics, and genomics, with spatial context, providing unparalleled insights into the organization, function, and interactions of cells within tissues. The methodologies of various spatial omics techniques are summarized in Table 1.

**Table 1.** Summary of the commonly used method of spatial omics technologies

Method	Year	Company	Principle	Sample requirement	Species compatibility	Throughput	Capture areas	Resolution	Advantages	Refs.
Spatial transcriptomics										
Tomo-seq	2014	-	Sequencing-based	Fresh frozen	Various	~12,000 genes per slide	Multiple 2D slices combined into 3D maps	-	Identifies genes expressed in spatial patterns	[28]
Visium	2016	10x Genomics	Sequencing-based	Fresh frozen, fixed frozen, FFPE	Various	20,000–30,000 genes per slide	6.5 mm × 6.5 mm	~100 μm	Simultaneous analysis of molecular and imaging data from FFPE & frozen tissue sections	[17]
GeoMx	2019	NanoString	Imaging- and sequencing-based	Fresh frozen, fixed frozen, FFPE	Various	Up to 18,000 genes per region	50–600 μm in diameter/ROI	~200 μm	Covers ~18,000 genes and supports whole transcriptome expression analysis on paraffin or fresh samples	[27]
MERSCOPE	2021	Vizgen	Imaging-based	Fresh frozen	Various	100–1000 genes per sample	20 mm × 20 mm	Subcellular level	High specificity and sensitivity; suitable for intercellular interaction studies	[21]
Esper	2021	Rebus Biosystems	Imaging-based	Fresh frozen	Various	Hundreds to thousands of RNA	20 mm × 20 mm	Single-molecule (~10 nm)	Ultra-sensitive method capable of detecting individual RNA molecules in situ	[22]
CosMx	2021	NanoString	Imaging-based	Fresh frozen, fixed frozen, FFPE	Various	Up to 1,000+ targeted genes per run	100 μm^2^ -1 cm^2^/ROI	Subcellular level	Enables high-resolution spatial analysis of complex tissue structures	[23]
STOmics	2021	BGI	Sequencing-based	Fresh frozen, fixed frozen, FFPE	Various	Thousands of genes per run	6 cm × 6 cm	~10 μm	Cost-effective platform provides high throughput for large-scale spatial transcriptomics studies	[30]
Xenium	2022	10x Genomics	Imaging-based	Fresh frozen, FFPE	Human and mouse	Up to 5000 genes per slide	Up to 472 mm^2^ of tissue	Subcellular level	Better able to spatially resolve and discriminate cell types than other Spatial Transcriptomics methods	[18–20]
Stereo-seq	2022	BGI	Sequencing-based	Fresh frozen	Various	300–500 genes per cell	13.2 cm × 13.2 cm	Nano resolution (~500 nm)	Achieves ultra-high resolution suitable for organ-level spatial analyses	[31]
Visium HD	2024	10x Genomics	Sequencing-based	Fresh frozen, fixed frozen, FFPE	Various	20,000–30,000 genes per slide	6.5 mm × 6.5 mm	Single-cell level (~2 μm)	Proven whole transcriptome spatial analysis at single cell-scale resolution and continuous tissue coverage	[32]
Spatial proteomics										
GeoMx DSP	2019	NanoString	Antibody-dependent spatial MS	Fresh frozen, FFPE	Various	Up to 96 proteins	600 μm × 600 μm/ROI	Single-cell level	Enables region-specific profiling without sample destruction	[46–48]
CODEX	2019	Akoya Biosciences	Cyclic antibody imaging-based	Fresh frozen, FFPE	Human and mouse	40–100 antibodies	Entire tissue sections	Single-cell level	Superior single-cell spatial resolution; cost-effective and scalable for large panels	[50]
MICS	2020	Miltenyi Biotec	Cyclic antibody imaging-based	Fresh frozen	Various	200+ proteins	14 mm × 14 mm	Subcellular level (~100 nm)	Capable of detecting rare cell populations and cell–cell interactions in situ	[49]
DISCO-MS	2021	-	Unbiased MS-based	Fresh frozen, fixed frozen	Human and mouse	-	Whole specimens	Subcellular level	Provides true 3D proteomics	[53]
PCF	2022	Akoya Biosciences	Cyclic antibody imaging-based	Fresh frozen, FFPE	Various	100+ antibodies	Entire tissue sections	Single-cell level (~0.25 μm)	Combines ultra-high multiplexing and single-cell resolution for entire tissue sections	[51,52]
Spatial metabolomics										
MALDI 2 tims-TOF FLEX	2020	Bruker	MALDI-MS	Fresh frozen	Various	-	Up to 10 cm^2^	~5 μm	High ionization and detection sensitivity; suitable for detecting over 800 metabolites	[71]
AFADESI-MSI	2019	-	DESI-MSI	Fresh frozen	Various	-	150 mm × 100 mm	20–100 μm	Nondestructive, high sensitivity, broad metabolite coverage, and suitable for complex tissues	[72]
SpaceM	2021	-	Fluorescence microscopy and MALDI-MS	Fresh frozen	Various	-	400 μm × 400 μm	Single-cell level (~1.5 μm)	High sensitivity, single-cell resolution, nondestructive imaging, and capability to study cellular metabolic heterogeneity	[73]
Spatial epigenomics										
Spatial-ATAC-seq	2022	-	Tags open chromatin with Tn5 transposase on tissue sections, combines spatial barcoding and sequencing to analyze chromatin accessibility	Fresh frozen	Human and mouse	-	-	~20 μm	Provides spatial information on chromatin accessibility, enabling regulatory insights	[80]
Spatial-CUT&Tag	2022	-	Uses antibodies to detect histone modifications on tissue sections, integrates spatial barcoding, and sequences for profiling	Fresh frozen	Human and mouse	9735 (H3K27me3); 3686 (H3K4me3)	-	~20 μm	Allows high spatial resolution histone modification analysis	[81]
Epigenomic MERFISH	2022	-	Combines MERFISH with epigenomic profiling to spatially map chromatin states	Fresh frozen	Mouse	Modifications on ~270 bases	-	Single-cell level (<1 μm)	Provides single-cell spatial epigenomic data and enables highly multiplexed analysis	[82]
Spatial genomics										
Slide-DNA-seq	2021	-	Places spatially barcoded beads on slides, overlays tissue sections to capture DNA sequences	Fresh frozen, fixed frozen	Various	-	Entire tissue sections	~10 μm	Preserves tissue structure and suitable for large-scale tissue sample analysis	[84]
IGS	2020	-	Combines ISS with standard NGS to simultaneously analyze DNA sequences and structures	Fixed frozen	Various	-	-	400–500 nm	Enables spatial mapping of thousands of genomic loci within individual nuclei	[85]

#### Spatial transcriptomics: Principles and applications

Spatial transcriptomics is a branch of spatial omics, focusing on mapping gene expression on tissue sections to reveal the spatial distribution and function of cells under specific physiological or pathological states [14]. Although traditional transcriptomics techniques can provide rich gene expression information, they fail to retain the spatial structural information of the sample. Furthermore, scRNA-seq, despite their high-throughput capture of transcription information at the single-cell resolution, overlook spatial information [15]. Furthermore, the emergence of spatial transcriptomics, particularly the development of in situ capture bulk sequencing technologies, not only preserves spatial information but also achieves high-speed, high-throughput analysis of gene expression, offering new perspectives for the study of intercellular interactions and tissue structural function [16]. The principles and workflow of spatial transcriptomics are shown in Fig. 1A.

In 2016, Ståhl et al. [17] pioneered the use of next-generation sequencing (NGS) to read the transcriptome, marking a milestone study in spatial transcriptomics. The research focused on spatially resolved quantitative gene expression data, enabling visualization of mRNA distribution within tissue sections. This was achieved by placing tissue sections on an array of reverse transcription primers, each with a unique positional barcode, which facilitated the presentation of high-quality RNA-seq data from human breast cancer, preserving 2-dimensional (2D) spatial information. Tissues containing mRNA are suitable for spatial transcriptomics, which means that applications of spatial transcriptomics are broad.

##### Imaging-based spatial transcriptomics technologies

Imaging-based technologies, such as Xenium, offer subcellular resolution, providing unprecedented detail in localizing RNA molecules within the cellular architecture. Its principle involves using circularizable DNA probes for amplification to localize a large number of target genes, suitable for further exploration after identifying target genes of interest [18,19]. This approach is crucial for studies requiring precise mapping of gene expression at the cellular or even subcellular level [20]. Technologies like the MERSCOPE 1000 Gene Panel by Vizgen allow for in situ multiplex gene detection, which is essential for analyzing the spatial distribution of different cell types and understanding intercellular interactions with high sensitivity and specificity [21]. Additionally, the Esper spatial omics platform developed by Rebus Biosystems employs single-molecule fluorescence in situ hybridization (smFISH) technology, facilitating the in situ analysis of individual RNA molecules and generating detailed gene expression images [22]. Additionally, the imaging-based CosMx Spatial Transcriptomics platform employs high-resolution imaging techniques to spatially map gene expression within tissue samples [23]. Despite their high resolution, these imaging-based methods are generally characterized by lower throughput, which can be a limiting factor when broad tissue analysis is required [24].

##### Sequencing-based spatial transcriptomics technologies

Conversely, sequencing-based methods such as Visium by 10x Genomics integrate microscopy imaging with RNA sequencing to link mRNA expression data from cells to their corresponding locations within tissue sections [25]. This method effectively captures transcriptome data across entire tissue sections, although it typically offers lower resolution compared to imaging-based techniques [14]. Specifically, the Visium platform features a spot size of 55 μm and a center-to-center distance of 100 μm between sample spots [26]. This spatial arrangement results in considerable portions of the tissue remaining unanalyzed for gene expression, thereby limiting the technology’s resolution and preventing it from providing single-cell level detail. Similarly, the GeoMx Spatial Profiling system from NanoString, based on micro-dissection technology, enables whole transcriptome analysis from single tissue sections, supporting the study of up to 18,000 protein-coding genes [27]. Another notable method, Tomo-seq, provides spatially resolved transcriptomic data by sequencing RNA from sequentially sectioned tissues, which helps map gene expression in 3D patterns without the need for manual image annotation [28]. By extracting RNA from individual sections and utilizing RNA sequencing, Tomo-seq achieves a resolution equivalent to single-cell analysis, effectively recapitulating gene expression within a 3D spatial context [29]. In addition, the sequencing-based STOmics Spatial Transcriptomics platform utilizes advanced sequencing technologies to precisely capture and analyze transcriptomic information in its spatial context [30]. Recent innovations such as Stereo-seq and the upgraded Visium HD have further advanced sequencing-based spatial transcriptomics by achieving single-cell resolution, enhancing both the detail and throughput of spatial gene expression analyses [31,32]. The Visium HD technology, in particular, theoretically supports resolutions up to 2 μm, potentially offering single-cell level resolution [33]. However, further research is needed to confirm that this level of detail is consistently achievable across various cell types and tissues. These advancements represent crucial progress in the field of spatial transcriptomics, addressing previous limitations and paving the way for more precise and comprehensive gene expression mapping.

The choice between imaging-based and sequencing-based spatial transcriptomics technologies often depends on the specific research question, where the required resolution and throughput dictate the appropriate method. While imaging-based methods offer finer detail at a lower throughput, sequencing-based approaches provide broader coverage with the ability to profile extensive tissue sections. Each method has its strengths and limitations, and the ongoing development in these technologies continues to improve their capabilities, making them increasingly indispensable in the exploration of complex biological systems.

Despite the important advantages of spatial transcriptomics, its application faces several challenges. For example, the number of features measured at each location by spatial transcriptomics is limited, with restricted measurement depth and throughput, difficulty in achieving single-cell resolution, high costs, and non-negligible sample heterogeneity [34]. Therefore, it is clear that spatial transcriptomics needs further development. Future efforts need to focus on improving resolution, simplifying sample processing workflows, and developing efficient data analysis tools [35]. For example, the development of 3D spatial omics technologies for high-precision measurement of entire organs, or the combination with multi-omics methods and other single-cell analysis technologies to measure DNA, RNA, and proteins simultaneously, could further enhance resolution and measurement accuracy, achieving a more comprehensive and precise analysis of disease mechanisms [34].

Currently, spatial transcriptomics technologies have been widely applied in various medical fields and have shown prominent potential in the study of cardiovascular diseases [36,37]. Spatial transcriptomics can provide unbiased spatial composition information, essential for creating precise physiological or pathological maps, which is crucial for understanding the spatial dynamics of heart development. Additionally, this technology can reveal the molecular mechanisms behind cardiovascular diseases, including the interactions between the cardiovascular disease microenvironment and cells, as well as the spatial distribution of molecular features in pathological tissues. Spatial transcriptomics also aids scRNA-seq in identifying new drug targets, offering new strategies for the treatment of cardiovascular diseases [38]. Although spatial transcriptomics has shown great potential in cardiovascular disease research, current studies mostly focus on validating scRNA-seq results, with relatively few cases utilizing NGS and other in situ capture techniques to delve into spatial information. Nonetheless, spatial transcriptomics holds remarkable scientific value and application prospects in the cardiovascular field. With continuous technological advancements and optimizations, spatial transcriptomics is expected to play an increasingly important role in the diagnosis, treatment, and prevention of cardiovascular diseases in the future.

#### Spatial proteomics: Insights into protein distribution

Proteins are the substances in the human body that directly exercise biological functions, and spatial analysis techniques based on proteins constitute spatial proteomics. Spatial proteomics captures the spatial information of proteins and studies their impact on cell function and potentially subcellular localization, although challenging, providing new evidence for revealing the complexity of cell functions and the dynamic evolution of tissue cellular structures [39–41]. The principles and workflow of spatial proteomics are shown in Fig. 1B.

The technical principles of spatial proteomics originated from immunohistochemistry (IHC) and in situ hybridization (ISH) methods that appeared half a century ago, but their resolution falls far short of the requirements of modern biological analysis techniques [34,42]. Current spatial proteomics technologies primarily rely on mass cytometry (MC) [43], which can be divided into imaging mass cytometry (IMC) [44] and multiplexed ion beam imaging (MIBI) [45] based on imaging principles. According to the use of antibodies, these technologies can be categorized into 2 types: antibody-based targeted spatial proteomics and mass spectrometry-based unbiased spatial proteomics (without predefined targets). Antibody-based targeted spatial proteomics are more widely used at present. These methods involve labeling antibodies with metals, fluorescent groups, DNA oligonucleotide barcodes, or special enzymes to target and locate specific proteins. Additionally, antibody-based targeted spatial proteomics is further subdivided into antibody-dependent spatial mass spectrometry techniques (GeoMx) and cyclic antibody imaging techniques (CODEX and MACSima). Cyclic antibody imaging, with its greater data generation capacity, allows for the detection of hundreds of proteins in situ at single-cell resolution [34]. It is particularly suitable for large sample volume studies using formalin-fixed paraffin-embedded (FFPE) or fresh frozen samples [34]. In contrast, mass spectrometry-based unbiased spatial proteomics does not require predefined target proteins, making it ideal for exploratory studies. However, their spatial resolution is often lower than targeted methods. Furthermore, since the expression of mRNA and its homologous proteins is nonlinear and highly regulated, the expression of mRNA cannot directly predict protein expression. Therefore, spatial proteomics can more accurately reflect the actual function and state of cells. However, despite its many advantages, subcellular resolution in spatial proteomics, while possible, remains challenging due to limitations in sensitivity, resolution, and the potential loss of spatial information during sample preparation [39].

Despite the application of spatial proteomics being less widespread than spatial transcriptomics, numerous spatial proteomics platforms have been developed. The GeoMx Digital Spatial Profiler (GeoMx DSP) by NanoString, launched in 2019, is a proteomics platform that uses nucleic acid probe-conjugated antibodies to capture target proteins in situ, allowing for the co-analysis of hundreds of proteins on the same paraffin tissue section [46,47]. DSP technology eliminates spectral overlap issues in traditional multiplex analyses and does not require enzymatic reactions such as nucleic acid amplification, thus improving target throughput and accuracy [48]. Around the same time, the MACSima Imaging Cyclic Staining (MICS) technology, developed by Miltenyi Biotec, has emerged as a pivotal tool in the proteomics landscape, enabling detailed analysis of spatial protein expression at the single-cell level [49]. The MACSima platform achieves single-cell resolution with a spatial precision of approximately 100 nm and supports the detection of over 100 antibodies, facilitating high-throughput analysis of tissue samples through both multiplexed imaging and mass spectrometry. Complemented by the innovative MACS iQ View software, this platform facilitates comprehensive exploration of image stacks generated by the instrument. MACS iQ View allows for seamless navigation through image stacks, segmentation of tissue images into single cells, and clustering based on cellular expression profiles. By visualizing and quantifying thousands of proteins, MACSima unveils intricate protein interactions and functional networks within tissue contexts, thereby offering profound insights into the spatial organization and molecular architecture of complex biological systems. Akoya Biosciences has introduced the PhenoCycler-Fusion platform (PCF; formerly CODEX [50]), a next-generation spatial proteomics technology based on multiplexed antibody fluorescence imaging [51]. CODEX enables single-cell resolution with a spatial precision of approximately 20 μm, allowing for the quantification of up to 100 protein markers across entire tissue sections [50]. In contrast, the latest PhenoCycler-Fusion 2.0 platform supports over 100 biomarkers and achieves an enhanced spatial resolution of 0.25 μm [51,52]. This improvement in resolution facilitates more detailed and precise spatial proteomic analyses, thereby advancing the capabilities of multiplexed protein imaging in biomedical research. The novel mass spectrometry-based spatial proteomics technology DISCO mass spectrometry (DISCO-MS) allows for unbiased spatial proteomic analysis of entire tissue regions, preserving their 3D spatial context and characterizing spatial molecular landscapes [53]. The new-generation Orbitrap Astral mass spectrometer features high sensitivity, analytical efficiency, and coverage depth, enabling reliable, high-throughput characterization of single cells and identification of various proteins in different phenotypic cells from complex samples [54–56]. Additionally, the new 4D proteomics platform spatial data-independent acquisition (DIA), based on timsTOF Pro and combined with laser capture microdissection (LCM) technology, accurately attains spatially specific cell populations, presenting spatial microscale molecular landscapes with high precision, high repeatability, and an average detection depth of over 2,000 proteins.

Spatial proteomics is currently widely applied in fields such as cell biology, pathology, drug discovery, and personalized medicine. Spatial proteomic analysis of organs or tissues can also reveal the spatiotemporal changes in organ development and map spatial protein landscapes [57]. Additionally, distinguishing cells with specific phenotypes enables exploration of the tumor microenvironment and heterogeneity [58] and a comparison of different cell types and states in situ, combined with captured spatial information, means that disease-related targets and biomarkers can be located [59].

#### Spatial metabolomics: Mapping metabolic activities

Metabolites are chemical substances produced by the metabolism of organisms, and a reflection of the physiological or pathological state of an organism can be obtained through the study of its levels and distribution characteristics. Spatial metabolomics is a molecular imaging technique based on mass spectrometry imaging (MSI) [60–63], which, compared to traditional metabolomics [64], can simultaneously obtain the molecular structure, content, and spatial distribution information on endogenous and exogenous metabolites from biological tissues. This enables the high-resolution spatial localization of thousands of metabolites within biological tissues, and the discovery of differential metabolites in situ, which also enables the identification of biological functions. This reveals metabolite distribution patterns and interrelationships in different spatial dimensions. This has important implications for the study of metabolic heterogeneity, accumulation, and regulation in tissues, organs, or certain substructures [65]. The principles and workflow of spatial metabolomics are shown in Fig. 1C.

The current commonly used platforms/software/databases for spatial metabolomics include METASPACE [66], MSiReader [67], OpenMSI [68], and MetaboLights [69]. Spatial metabolomics platforms are mainly based on mass spectrometry technologies, including desorption electrospray ionization MSI (DESI-MSI) and matrix-assisted laser desorption/ionization MSI (MALDI-MSI), which enable qualitative and quantitative analysis of metabolites with high spatial resolution [70]. MALDI-MSI is the most widely used mass spectrometry technology in spatial metabolomics, with the Bruker MALDI 2 tims-TOF FLEX spatial MSI platform achieving resolutions as high as 5 μm and high ionization and detection sensitivity capable of detecting over 800 metabolites [71]. The air flow-assisted desorption electrospray ionization MSI (AFADESI-MSI) spatial metabolomics technology can detect various spatial resolutions and is more conducive to qualitative analysis of lipids and small-molecule metabolites [72]. The SpaceM spatial metabolomics platform consists of secondary ion mass spectrometry and data analytics tools that facilitate detection of metabolic characteristics at each spatial position in cryo-sectioned tissue slices through high-resolution mass spectrometry analysis, yielding single-cell level metabolic profiles after algorithm processing [73].

As an emerging biotechnology, spatial metabolomics has been widely applied in various systems of animals and humans. Spatial metabolomics enables the exploration of the spatial distribution characteristics of metabolites in biological tissues and their pathogenic mechanisms, with a focus on tumor metabolism development methods and tumor diagnostic markers. Spatial metabolomics has been researched in areas such as tumorigenesis [74], colorectal cancer [75], and esophageal cancer [76].

#### Spatial epigenomics and spatial genomics: Emerging technologies

Understanding the organization and dynamic changes of epigenetic and genetic information within 3D spaces, as well as their impact on cell behavior and function, is crucial for normal biological development, tissue regeneration, and disease progression [77,78]. Both spatial epigenomics and spatial genomics are emerging branches of spatial omics that combine high-throughput analysis techniques with spatial resolution imaging to understand the organization and function of the epigenome and genome at tissue, organ, and cell level.

Spatial epigenomics involves the spatial analysis of epigenetic information (such as chromosome structure and epitopes, chromatin accessibility, DNA methylation, histone modifications, etc.) in tissues [79]. Key spatial epigenomics methods include spatial-ATAC-seq, spatial-CUT&Tag, and Epigenomic MERFISH. Spatial-ATAC-seq is a method for analyzing chromatin accessibility with spatial resolution in tissue sections, which can depict tissue region-specific epigenetic landscapes, enhancing understanding of cell identity, state, and fate determination, and promoting the development of spatial biology and the epigenetic basis of diseases [80]. With a spatial resolution of 20 μm/pixel, spatial-ATAC-seq is capable of resolving certain cell types, such as a thin layer of notochord cells, although it may not capture all cell types within a tissue [80]. Other technologies, such as spatial-CUT&Tag, allow for spatially resolved whole-genome analysis of histone modifications [81]. This method has been used to reveal the epigenetic control of the spatial patterns of cell types and development in brain cortex of mice, leading to developments in areas such as epigenetic regulation, cell function, and fate determination in normal physiology and disease mechanisms. Single-cell epigenome resolution can be obtained in situ by identifying 20 μm/pixels that contain a single nucleus through immunofluorescence imaging [81]. Furthermore, Epigenomic MERFISH can be used for spatially resolved single-cell epigenomic mapping, showing the subnuclear distribution of epigenetic loci, deepening the understanding of spatiotemporal gene expression regulation by the epigenome [82]. The principles and workflow of spatial epigenomics are shown in Fig. 1D.

The definition of spatial genomics has been subject to some ambiguity. According to Bouwman et al. [83], the term spatial genomics is suggested to describe the spatial arrangement of genetic material within the cell nucleus, emphasizing the 3D organization and structure of chromosomal interactions. On the other hand, Zhao et al. [84] describe spatial genomics as the study of gene expression and genomic features while preserving spatial context in tissues, allowing for the mapping of gene activity and molecular interactions across cellular locations. This variation in definitions underscores the complexity and broad scope of spatial genomics, encompassing both genome structure and spatially resolved gene activity.

Furthermore, spatial genomics relies on high-throughput sequencing technologies and in situ genome sequencing (IGS) is one of the key methods used. However, its resolution and sequencing depth need further improvements [85]. Another method is Slide-DNA-seq, which captures DNA sequences and spatial information in situ from tissue sections [84]. Spatial genomics has found relatively broad applications in the field of oncology, including cancer diagnosis and detection of tumor heterogeneity [77,84,86]. The principles and workflow of spatial genomics are shown in Fig. 1E.

However, due to the limited profiling depth and resolution of spatial epigenomics and spatial genomics, research in the cardiovascular field using these emerging methods is scarce. However, it is foreseeable that with advancements in sequencing technologies, these methods will become important tools for studying cardiovascular diseases.

#### Latest fields: 3D and 4D omics

Unlike 2D approaches that treat tissues or cells as flat structures, 3D omics incorporates the whole spatial architecture of tissues and organs, providing deeper insights into the dynamic interactions between cells, their microenvironments, and disease processes. This approach is particularly valuable for understanding complex phenomena such as cardiovascular systems, tumor microenvironments, brain structures, and developmental processes, where cellular context is crucial for function and disease progression. Advanced technologies enabling 3D omics include sequential sectioning and imaging methods, such as 3D MALDI MSI, which reconstructs 3D datasets from consecutive 2D sections [87]. SpatialScope integrates spatial and single-cell transcriptomics to create detailed 3D maps of tissues, such as the mouse brain cortex [88]. Additionally, recent advancements have introduced sequencing-based, open-source experimental and computational resources that offer high-resolution, cost-efficient, and scalable 3D spatial transcriptomic analyses. Open-ST successfully captured transcripts at subcellular resolution in the mouse brain, reconstructed diverse cell types in primary head-and-neck tumors and patient-matched lymph nodes, and revealed spatially contiguous structures and potential biomarkers at the 3D tumor/lymph node boundary—insights that are not achievable with traditional 2D methods [89]. Furthermore, Qiu et al. [90] presents innovative frameworks for integrating temporal dynamics with spatial molecular data, enabling the visualization and analysis of how molecular interactions evolve over time within 3D contexts [90]. Tools like Spateo, which offer versatile 3D spatiotemporal modeling and interactive visualization for whole-embryo datasets, exemplify the ongoing efforts to enhance 3D omics capabilities.

Emerging interests in 4D omics aim to incorporate the temporal dimension, allowing researchers to observe how biological systems change over time in 3D space by combining time-lapse imaging with multi-omics data integration. An in-house light-sheet microscope and customized computational analysis enable high-resolution 4D imaging of its contracting heart in zebrafish at single-cell resolution [91]. By incorporating microinjection of small molecules to induce cardiac injury in a controlled manner, researchers can investigate both physiological and pathophysiological changes that shed light on the mechanisms of cardiac morphogenesis and regeneration. This highlights the need for continued innovation and exploration in these emerging fields to unlock their full potential in understanding and treating complex biological systems and diseases. However, further technological advancements are essential to fully realize 4D omics. Due to current technical limitations, it is challenging to obtain continuously sampled temporal data using traditional spatial omics approaches. A potentially feasible method involves conducting 3D omics analyses at different time points (for example, at various developmental stages of the same species or across different stages of disease progression within the same organ) and subsequently developing artificial intelligence (AI) algorithms capable of aligning 3D omics datasets and temporally integrating data obtained from discrete time points. Unfortunately, research into the applications of 4D omics in areas such as cardiovascular development, cardiovascular aging, and disease progression remains exceedingly limited.

### Sample preparation for spatial omics technologies

Sample preparation is a critical aspect of spatial transcriptomics and involves distinct methodologies based on tissue type and preservation. FFPE, fresh frozen, and fixed frozen tissues provide unique strengths and limitations in the realm of spatial omics research, influencing their application in spatial omics research. Understanding these preservation methods, along with their pros and cons, is essential for selecting the most appropriate approach to meet specific research needs and achieve standardized, reproducible outcomes. The comparative characteristics of these sample preparation methods are summarized in Table 2.

**Table 2.** Comparative summary of sample preparation for spatial omics technologies

Characteristics	Fixed frozen	Fresh frozen	FFPE
Fixed or not	Yes (using chemical fixation)	No	Yes (formalin fixation followed by paraffin embedding)
Molecular preservation	Partially modified	Closest to original state	Modified with potential molecular degradation (e.g., RNA)
Morphological quality	High	High (requires proper storage methods)	Excellent (suitable for long-term pathological observations)
Degradation risk	Low	High (prone to improper storage)	Very low
Storage duration	Medium-term (2–5 years)	Short-term (months to 2 years, requires low-temperature storage)	Long-term (decades at room temperature)
Common techniques	Spatial transcriptomics (DBiT-seq, Xenium)	Spatial transcriptomics (Visium, RNA-Rescue SRT)	Spatial transcriptomics (GeoMx, RNA panels for FFPE)
	Spatial proteomics (imaging mass cytometry)	Spatial metabolomics (MALDI-MSI, DESI-MSI)	Specialized spatial proteomics (GeoMx DSP, tandem mass tagging)
	Spatial epigenomics (spatial-ATAC-seq)	Spatial epigenomics (spatial-CUT&Tag)	Long-term storage suitable for retrospective studies
Advantages	Preserves tissue structure, compatible with some omics analyses	Molecular information well-preserved, ideal for high-throughput analyses	Excellent morphological preservation, ideal for archival studies
Disadvantages	Chemical fixation may hinder molecular experiments	Requires freezing, prone to degradation	Considerable challenges for RNA-based measurements
Usage frequency	Moderate	High	Very high

#### FFPE: Ideal long-term morphology preservation

The standard procedure for preparing FFPE samples involves tissue fixation, where surgical specimens are immersed in a formalin solution (typically 4% formaldehyde). Following fixation, tissues are dehydrated through sequential alcohol baths, cleared with xylene, and embedded in paraffin wax to create stable blocks. These paraffin blocks are sectioned into thin slices, usually 5 μm thick, using a microtome, mounted on slides, stained for histological analysis, and stored for long-term use [92].

FFPE preparation offers unparalleled long-term stability, allowing tissue samples to be archived for years without obvious degradation, making it ideal for retrospective studies. This enables researchers to analyze rare or unique tissue samples, such as archival cancer biopsies, which are invaluable for longitudinal studies and biomarker discovery. Additionally, FFPE tissues are compatible with IHC staining, enabling detailed visualization of tissue morphology and protein localization. However, the fixation process introduces molecular alterations, such as chromatin damage from prolonged formaldehyde exposure and protein cross-linking, which can hinder protein extraction and complicate proteomic and epigenomic analyses [93].

Innovative strategies like the cleavage under targeted accessible chromatin (CUTAC) protocol address these challenges by yielding small fragments robust to DNA degradation, enabling reliable profiling of FFPE samples [94]. Similarly, Faktor et al. [92] demonstrated the effectiveness of tandem mass tag (TMT) labeling and booster channel techniques for improving the resolution of proteomic analyses in FFPE tissue subsections.

#### Fresh frozen: Optimal molecular integrity

Fresh frozen samples are widely regarded as the gold standard for unbiased polyA-based spatial transcriptomics technologies due to their exceptional preservation of polyadenylated transcripts. To prepare fresh frozen samples, freshly excised tissues are rapidly frozen using liquid nitrogen or isopentane cooled with dry ice, ensuring the preservation of RNA, DNA, proteins, and metabolites in their native states. This approach minimizes molecular degradation, making it suitable for advanced spatial omics techniques such as transcriptomics, proteomics, and metabolomics.

Despite these advantages, fresh frozen preparation poses logistical challenges, as maintaining the frozen state requires ultra-low temperature storage and specialized handling protocols to prevent thawing and degradation. To address the limitations of degraded samples, RNA-rescue spatial transcriptomics (RRST) was developed. RRST uses a gene panel initially designed for FFPE samples but includes additional modifications such as gentle formalin fixation and a baking step to optimize fresh frozen tissues for spatial transcriptomics [95]. Furthermore, Liao et al. [96] introduced the spatial protein and transcriptome sequencing (SPOTS) method, which simultaneously detects mRNAs and proteins with high spatial resolution and reproducibility in fresh frozen tissues. Martínez et al. [97] utilized fresh frozen skeletal muscle tissues in spatial metabolomics, revealing regionalized gene expression, metabolic variations, and differences in myofiber types along the proximal–distal axis.

#### Fixed frozen: Balanced preservation

Fixed frozen tissue preparation combines the benefits of fixation and rapid freezing to preserve both spatial and molecular integrity, making it an emerging choice in spatial omics research. The protocol typically involves formalin or paraformaldehyde fixation, followed by freezing to maintain tissue structure and molecular composition [98]. This method is highly compatible with advanced spatial omics techniques, including spatial transcriptomics and proteomics. For example, Xenium spatial transcriptomics has utilized fixed frozen mouse brain sections to achieve high-resolution gene expression mapping while preserving RNA integrity [99]. Similarly, spatial multi-omics sequencing via DBiT-seq integrates transcriptomics and proteomics in fixed frozen tissues, enabling multimodal profiling at subcellular resolution [98]. Fixed frozen tissue also excels in preserving tissue morphology and antigenicity, crucial for IHC and MSI.

#### LCM facilitates the precise resolution of spatial locations

LCM is a method used to obtain spatial information from regions of interest (ROIs) within tissues, with a resolution that can approach single-cell levels. LCM has been applied to study localized gene expression changes during the progression of atherosclerosis, although it is prone to contamination by RNA from adjacent nontarget cells [100]. Currently, many mass spectrometry-based spatial omics techniques rely on LCM to acquire ROIs. However, these methods often struggle to achieve high sample throughput and multiplex sequencing, and they demand advanced instrumentation and imaging systems, which limits the widespread adoption of this technology in spatial omics research [101].

### Diverse tools for spatial omics data analysis

Technological advances in spatial omics have enabled a deeper understanding of complex biological systems; however, deriving meaningful insights requires robust data analysis and computational tools designed to address the complexity inherent in spatial data. Tools like Squidpy offer a flexible and scalable framework for analyzing spatial molecular data, particularly in intricate tissue structures [102]. By leveraging graph-based analysis, Squidpy facilitates data exploration, clustering, and visualization, making it possible to decode cell–cell interactions and spatial heterogeneity within tissues. Its key features, such as neighborhood enrichment analysis, spatial autocorrelation, and molecular co-expression analysis, make it a valuable asset for spatial transcriptomics studies [102].

Seurat, a versatile tool well-known for its single-cell analysis capabilities, has extended its functionality to spatial omics with a dedicated spatial transcriptomics module. This allows for spatially resolved clustering, integration with single-cell RNA-seq data, creation of spatial feature plots, and overlaying of gene expression data on tissue images, offering a comprehensive platform for exploring spatial heterogeneity [103]. Giotto also stands out as a comprehensive tool tailored for analyzing and visualizing spatial transcriptomic data. Its capabilities include multiscale clustering, spatial network construction, 3D rendering of spatial data, and support for a variety of spatial omics formats, providing a holistic approach to examining spatially resolved datasets [104].

In addition to these tools, innovative platforms and algorithms have emerged to integrate multi-omics data within spatial contexts. SpatialGlue employs a graph neural network model with a dual-attention mechanism to combine spatial multi-omics data from the same tissue section, focusing on integrating spatial locations and omics measurements for more accurate domain delineation [105]. The Spatial-Linked Alignment Tool (SLAT) addresses challenges such as nonrigid deformations and batch effects through graph adversarial matching, aligning heterogeneous spatial datasets across different technologies and experimental conditions with high accuracy and speed [106]. SpatialData is another framework that facilitates the integration and analysis of multimodal spatial omics datasets, supporting various spatial technologies and data types to enhance reproducibility and scalability [107]. The GENIUS framework transforms multi-omics data into images and applies deep learning models to extract key information, which has been used to predict disease progression and discover disease-associated genes [108].

Despite these tailored computational approaches, the integration of diverse datasets to provide a cohesive sample overview remains a prominent challenge in multi-omics. Argelaguet et al. [109] outlined 3 main types of integration tasks: horizontal integration, vertical integration, and diagonal integration. Horizontal integration applies the same analysis across different samples, while vertical integration focuses on different spatial omics layers within a single sample. However, comprehensive and standardized parallel measurements across spatial omic modules remain limited, especially when connections between datasets are ambiguous. Machine learning holds promise for addressing these challenges by uncovering hidden connections in complex datasets, as demonstrated by various AI-driven approaches [110].

While progress in spatial omics has been considerable, challenges in data integration, spatial resolution, and computational complexity persist, particularly when examining intricate tissues such as the heart. Effective integration of multimodal data types, management of batch effects, and the development of standardized analytical workflows are critical for ensuring reproducibility and scalability. Continued advancements in data integration frameworks, reproducible workflows, and improved algorithms are essential for driving spatial omics research forward, unlocking its full potential in precision medicine and cardiovascular science.

### Cardiac-specific analytical challenges

The heart is a highly complex and meticulously organized organ composed of diverse cell types, exhibiting distinct spatial heterogeneity [111]. However, most spatial omics platforms, such as Visium, are limited by the area of tissue they can analyze per slice (Table 1), thereby restricting the identification of extensive spatial spans and making it challenging to comprehensively present anatomically distant structures within a single section. Additionally, the essential role of the heart in sustaining human life, coupled with ethical concerns, makes the collection of normal heart samples exceedingly difficult. Consequently, most studies utilizing normal heart samples rely on autopsies or transplantation donors with size mismatches, and the sample sizes are typically very limited. This limitation somewhat restricts the application of spatial omics in the cardiovascular field. Furthermore, there are currently few high-resolution spatial omics reference maps of the human heart reported [112], which hampers the accurate identification and spatial localization of cell types, representing a major challenge for future research in this area.

## Spatial Transcriptomics in Cardiovascular Research

**Fig. 2.**
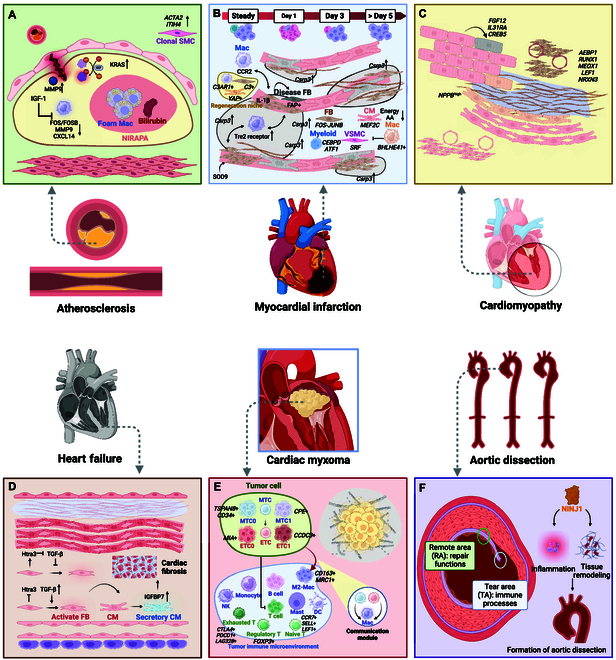
Schematic view of cardiovascular diseases using spatial transcriptomics. (A) Atherosclerosis: Showed high macrophage infiltration and molecular signatures related to plaque stability within atherosclerotic plaques. (B) Myocardial infarction: Displayed distinct gene expression patterns in infarcted and peri-infarct zones, highlighting molecular responses to ischemic injury. (C) Cardiomyopathy: Revealed fibrosis-related genes and altered cellular composition in HCM tissues. (D) Heart failure: Uncovered region-specific gene expression changes that contribute to the progression of HF. (E) Aortic dissection: Highlighted distinct molecular profiles in dissection sites, providing insights into pathophysiology and potential therapeutic targets. (F) Cardiac myxoma: Identified tumor-specific gene signatures and interactions within the tumor microenvironment. CM, cardiomyocyte; FB, fibroblast; Mac, macrophage; SMC, smooth muscle cell; ETC, EC-like tumor cells; MTC, MSC-like tumor cells. Created with http://BioRender.com.

The application of spatial transcriptomics has gained noteworthy attention in the cardiovascular domain, encompassing cardiac development, diverse cardiovascular disorders, the cardiac conduction system, drug assessment, surgical transplantation, and more [25]. It is plausible to anticipate that spatial transcriptomics will emerge as a pivotal research tool in the realm of cardiovascular studies. A schematic of spatial transcriptomics studies in cardiovascular disease is shown in Fig. 2, and a summary is shown in Table 3.

**Table 3.** Summary of spatial transcriptomics in the research of cardiovascular diseases

Disease	Species	Year	Platform	Key target	Primary discoveries	Refs.
Studies using human samples						
Atherosclerosis	Human	2023	10× visium	Marcophages	NIRAPA signals spatially coincided with bilirubin and inflammatory macrophages	[126]
Atherosclerosis	Human	2023	10× visium	Atherosclerotic plaques	MMP9 expressed more in areas of plaque rupture and promoted the incidence of plaque rupture	[128]
Atherosclerosis	Human	2023	Database	Atherosclerotic plaques	Three regional clusters within the plaque were identified as well as characteristics of inflammation and calcification	[129]
Atherosclerosis	Human	2023	10× visium	SMCs	The progression of atherosclerosis is accompanied by a slight proliferation of SMC populations	[131]
Atherosclerosis	Human	2024	GeoMx	SMCs	Identified transcriptomic differences between stable and unstable coronary plaques, emphasizing inflammation and SMC phenotypic changes	[133]
Atherosclerosis	Human	2024	10× visium	Atherosclerotic plaques	Complex cellular interactions were identified within the adventitial and subendothelial regions in human atherosclerosis.	[130]
MI	Human	2022	10× visium	Marcophages, FBs	The spatial dependence of MI between different cell niches was revealed	[136]
MI	Human	2023	Database	CMs	Fewer CMs survived in the infarcted heart, and energy and amino acid metabolic pathways were down-regulated in the surviving CMs, indicating a unique metabolic adaptation of CMs to survive after infarction	[137]
MI	Human	2023	10× visium	Marcophages, FBs	Interactions between CCR2^+^ macrophages and fibroblasts mediated by IL-1β signaling promote the differentiation of disease-associated fibroblasts into profibrotic cells	[138]
MI	Human	2024	Database	FBs	Mapped fibroblast heterogeneity and revealed key cellular transitions driving cardiac fibrosis	[139]
MI	Human	2024	10× visium, MERFISH	Nonimmune cell	uncovered spatially localized interferon-induced clusters at the injury border zones in myocardial infarction, driven by nonimmune cells via the IRF3 pathway	[144]
ARVC	Human	2023	Tomo-seq	CMs	ZBTB11 is specifically concentrated in active fibro-fatty replacement sites within the myocardium	[149]
HCM	Human	2023	10× visium	CMs	The CM subpopulation with high expression of NPPB is situated in close proximity to the fibrotic area	[152]
HCM	Human	2023	GeoMx	FBs, vascular cells	The composition of fibroblasts and vascular cells in the severely affected areas of HCM also exhibits significant differences	[153]
HF	Human	2017	10× visium	Deference of spatial gene expression	In HF patients with preserved ejection fraction, there is spatial variation in the expression of fetal marker genes expressed by the small cell subse	[155]
HF	Human	2024	10× visium	Right atrial tissues	Revealed proinflammatory microvascular changes and endothelial cell heterogeneity in ischemic heart disease	[157]
Cardiac myxoma	Human	2024	10× visium	MTCs, ETCs, macrophages	Cardiac myxoma tumor cells include endothelial-like tumor cells and mesenchymal stem cell-like tumor cells, with the former differentiating from the latter	[159]
TAD	Human	2023	Tomo-seq	Gene expression features from tear to distal area	NINJ1 may be involved in inflammation and tissue remodeling, playing a crucial role in the formation of TAD	[161]
Viral myocarditis	Human	2023	GeoMx	Deference of spatial gene expression	The spatial resolution of gene expression profiles from the epicardium to the endocardium provided insights into regional responses and remodeling processes during infection	[163]
Viral myocarditis	Human	2023	GeoMx	Marcophages	Up-regulation of genes associated with DNA damage and repair, heat shock, and infiltration of M1-like macrophages in cardiac tissues of patients	[164]
Cardiac sarcoidosis	Human	2022	GeoMx	CD68^+^ immune cell	Determining the spatial relationship of CD68^+^ immune cell infiltration in human cardiac sarcoidosis	[172]
Coarctation of the aorta	Human	2021	10× visium	Cellular populations in the arterial wall	Visualized the precise location of cell clusters within the arterial wall structure	[174]
Aneurysms	Human	2023	MERFISH	SMCs	The expression of CARTPT in the SMC subpopulation is enriched in characteristic spatial features, and human CART promotes the switch between bone and soft cartilage generation in aortic SMCs	[176]
Aneurysms	Human	2024	10× visium	VSMCs	Identified key regulatory dynamics in vascular smooth muscle cells associated with aortic root aneurysms	[177]
Atrial fibrillation	Human	2024	GeoMx	Resident memory T cell	Identified 2 transcriptionally distinct tissue-resident memory T cell subsets in epicardial adipose tissue contributing to atrial fibrillation pathophysiology	[178]
Studies using samples from other species						
Atherosclerosis	Pig	2023	10× visium	Marcophages	IGF-1 played important pathophysiological role in atherosclerotic processes	[127]
Atherosclerosis	Murine	2024	Resolve BioSciences (ISH)	SMCs	ITIH4 was specifically expressed in SMCs of atherosclerotic lesions	[132]
MI	Murine	2017	Tomo-seq	Cardiac fibrosis	SOX9 was a key regulator of cardiac fibrosis during MI	[135]
MI	Murine	2022	10× visium	Immune cells	Significant spatial and temporal heterogeneity of immune cell populations and macrophages was found during MI	[140]
MI	Murine	2022	10× visium	Border zone	Csrp3 was a potential therapeutic target for preventing ventricular remodeling post-MI	[141]
MI	Murine	2022	10× visium	CMs	There are significant regional isotypic switches between different transcripts of genes involved in myocardial contraction and tissue morphogenesis	[142]
MI	Murine	2023	10× visium	Marcophages	BHLHE41^+^ macrophages inhibited myofibroblast activation, prevent excessive fibrosis, and limit infarct expansion	[143]
MI	Murine	2024	10× visium	CMs	Conservative regenerative CM populations colocalize in space with cardiac fibroblasts expressing the C3 gene and macrophages expressing C3AR1, forming an ecological niche that promotes myocardial regeneration	[145]
MI	Murine	2024	10× visium	Gelsolin	Gelsolin plays an important role in ventricular remodeling following myocardial infarction	[146]
HF	Murine	2022	10× visium	CMs	The Htra3-TGF-β-IGFBP7 axis plays a crucial role in regulating CMs homeostasis, preventing myocardial fibrosis in the infarcted area, and inducing phenotypic secretion of CMs	[156]
Vasculitis	Murine	2021	10× visium	Cell communities in vascular tissues	The activation of the NLRP3 inflammasome pathway and the expression characteristics of key cell types in vasculitis were discovered	[170]
Pulmonary arterial hypertension	Murine	2024	10× visium	ECs	Pulmonary arterial hypertension leads to a transformation of capillary endothelium into arterial endothelium	[175]

### Applications in healthy cardiovascular system

#### Cardiac anatomical structure

With the advent and diversification of sequencing technologies and the provision of spatial context, single-cell level gene expression maps have provided new insights into the study of the cardiac anatomical structure. Kanemaru et al. [112] employed advanced spatial omics techniques, including scRNA-seq, snRNA-seq, snATAC-seq (single-nucleus assay for transposase-accessible chromatin sequencing), and spatial transcriptomics, to map the cellular and molecular architecture of the human heart. By analyzing tissues from 25 healthy heart transplant donors across 8 anatomical regions, the study identified distinct cell populations and their specific spatial distributions within various anatomical regions of the heart. By integrating transcriptomic and proteomic data, the research uncovered complex interactions and signaling pathways that maintain heart structure and function. Additionally, the study spatially characterized cells of the cardiac conduction system and identified clinically relevant cell states and microenvironment models. These findings provide valuable insights into the spatial organization of cardiac cells, enhancing our understanding of cardiovascular biology and the mechanisms underlying heart diseases. Hsu et al. [113], by combining embryonic high-resolution imaging, single-cell genomics, spatial transcriptomics, proteomics, and other methods, discovered that the intrinsic cardiac nervous system (ICNS) uses extracellular matrix genes that match those of surrounding cardiac cells, adopting an intermediate dedicated neuronal progenitor state to form unique structures on the atria. This reveals the fundamental principles of intrinsic neuronal maturation and specialization within the heart. Sun et al. [114], utilizing advanced single-cell and spatial transcriptomic analyses, identified a group of CMs in the mouse heart expressing the *DBH*, which encodes dopamine β-hydroxylase, and determined the distribution of these CMs within the heart, providing new perspectives on the development and heterogeneity of the mammalian cardiac conduction system.

#### Cardiovascular development

The heart, as the first organ to develop, has its function intrinsically tied to its morphology. However, human heart morphogenesis is not yet fully understood [115]. Uncovering the key molecules and signaling pathways involved in cardiac development could have profound implications for treating cardiovascular diseases. Asp et al. [116] provided foundational insights by creating a comprehensive transcriptional atlas of cell types during embryonic heart development. Through spatial transcriptomics, they identified unique gene signatures for different anatomical regions at each developmental stage, offering a vital overview of heart formation. Expanding on the role of CMs in heart development, Sylvén et al. [117] integrated scRNA-seq, spatial transcriptomics, and ligand–receptor interaction data. They identified 8 types of developing CMs with marked variability in cell cycle activity, mitochondrial content, and connexin gene expression. This high heterogeneity and location-dependent distribution of CMs in atria and ventricles underscores the complex regulation and differentiation of CMs in human heart formation. Cui et al. [118] further explored cardiac development by examining the role of the transcription factor NFYa using spatial and single-cell transcriptome analysis in mouse hearts. They found that the absence of NFYa altered CM composition, reducing proliferation and impairing mitochondrial metabolism, ultimately leading to heart growth defects. This study highlighted the essential role of mitochondrial metabolism in heart development and its implications for cardiac health. In addition to heart morphogenesis, the development of arterial valves also warrants focused investigation due to potential congenital defects, such as bicuspid aortic valves. Queen et al. [119] applied spatial transcriptomics to analyze the transcriptome of developing arterial valves, revealing that most differentially expressed genes were valve-specific, with RBP1 being a potential key player in valve development. Furthermore, Du et al. [120] used spatial transcriptomics to differentiate between the transcriptional profiles and functions of epicardial and non-epicardial tissues. They identified embryonic epicardial tissue-specific markers, including *UPK3B*, *EFEMP1*, *MSLN*, and C3, and demonstrated that extracellular matrix signaling is critical for epicardium maturation. This discovery opens new therapeutic targets for cardiac regeneration in future clinical applications. Building upon these insights, Farah et al. [121] combined scRNA-seq with high-resolution multiplexed error-robust fluorescence ISH to generate a spatial map at the single-cell level. Their findings highlighted cellular communities responsible for shaping different cardiac structures and revealed how cardiac cell subpopulations specialize according to their anatomical regions and cellular ecosystems. This work opens new avenues for understanding structural heart disease and engineering complex human heart tissues, emphasizing the critical nature of spatial perspectives in cardiac research. Lázár et al. [122] analyzed early human embryonic samples using scRNA-seq and spatial transcriptomics, providing a molecular and cellular atlas of human heart development and elucidating the developmental processes of the cardiac pacemaking-conduction system. Collectively, these studies illustrate the intricate processes driving cardiac development, highlighting the value of spatial and single-cell transcriptomics in understanding heart morphogenesis and identifying potential therapeutic interventions for cardiovascular diseases.

#### Cardiovascular aging

Aging is also expected to be a prominent research focus for spatial omics in the cardiovascular field in the future. Ma et al. [123] employed Stereo-seq to delineate the spatiotemporal atlas of aging across all organs in mice. They discovered that CMs exhibit significant alterations in the expression of genes related to inflammatory responses and energy metabolism dysregulation during the aging process, with ventricular muscle showing heightened sensitivity to aging. Additionally, the study revealed that the aging of cardiac tissue is accompanied by disrupted spatial architecture and the aggregation of immunoglobulin G (IgG) near CMs. These findings provide critical insights into the molecular and spatial dynamics of cardiac aging, highlighting potential targets for therapeutic intervention.

#### Cardiovascular immune niche

Due to the size limitations of CMs, single-cell level studies of CMs in cardiovascular system predominantly utilize snRNA-seq [9]. However, snRNA-seq has a lower capacity for capturing immune cells compared to scRNA-seq [124]. Consequently, the inherent immune niches within the cardiovascular field remain underexplored. By integrating spatially resolved multi-omic techniques, Kanemaru et al. [112] investigated immune niches within both healthy and diseased human hearts. Their research reveals a heterogeneous distribution of immune cells with specialized functions based on their spatial locations and demonstrates how alterations in these distributions contribute to the progression of cardiovascular disease.

### Applications in atherosclerosis

Atherosclerosis is a type of arterial stiffness characterized by the development of lesions in the arterial wall. These lesions may lead to narrowing of the arterial wall due to the accumulation of atherosclerotic plaques [125]. Schneider et al. [126] established the potential of combining near-infrared auto-photoacoustic (NIRAPA)-ultrasound imaging to detect vulnerable arterial atherosclerotic plaques. Utilizing IHC, spatial transcriptomics, and proteomics on adjacent plaque sections, they discovered that the highest NIRAPA signals spatially coincided with bilirubin and associated blood residuals, as well as with inflammatory macrophages. Previous studies have indicated that insulin-like growth factor 1 (IGF-1) may reduce coronary artery plaque burden and promote plaque stabilization in mouse models. Using spatial transcriptomics, Sukhanov et al. [127] investigated the overall transcriptomic changes in late-stage atherosclerotic plaque regions in pigs. Their analysis revealed that IGF-1 down-regulated the expression of FOS/FOSB factors, as well as *MMP9* and *CXCL14* genes in plaque macrophages, suggesting that these molecules are involved in the modulatory effects of IGF-1 on atherosclerosis. These findings provide further evidence for the pathophysiological role of IGF-1 in atherosclerotic processes. Sun et al. [128] unveiled the localization of *IGKC*, *MMP9*, and *PLN* within atherosclerotic plaques by spatial transcriptomics, identifying a proximal expression of MMP9 in these plaques. In this research, spatial transcriptomics was first used in human atherosclerotic plaques to detect the spatial distribution of *MMP9*-related gene expression and pathways. Results from the research indicated more significant *MMP9* expression in areas of plaque rupture and an up-regulation of allograft rejection and KRAS signaling pathways, promoting the incidence of plaque rupture [128]. Theofilatos et al. [129] conducted a proteomic analysis of carotid endarterectomy samples and integrated proteomic features with scRNA-seq and spatial RNA sequencing data to identify 3 regional clusters within plaques as well as characteristics of inflammation and calcification, revealing patient subgroups associated with the risk of cardiovascular events. Bleckwehl et al. [130] revealed the critical role of the microvasculature in human atherosclerosis by integrating spatial and scRNA-seq to create a high-resolution map of atherosclerotic plaques.

Furthermore, smooth muscle cells (SMCs) are strongly associated with the progression of atherosclerosis. Kawai et al. [131] employed RNA ISH to investigate whether populations of SMCs exist within human unstable atherosclerotic plaques and used spatial transcriptomics to identify differentially expressed transcripts within clonal and nonclonal areas. They discovered that the progression of atherosclerosis is accompanied by a slight proliferation of SMC populations. Ravindran et al. [132] utilized translating ribosome affinity purification sequencing to directly analyze the expression of SMC-specific genes from tissues. They confirmed the SMC-specific expression of *ITIH4* in atherosclerotic lesions through the immunofluorescence staining of mouse aortas and spatial transcriptomic analysis of human carotid arteries. Using GeoMx spatial transcriptomics platform with whole-transcriptome analysis to identify up-regulation of pro-inflammatory and pro-thrombotic pathways in human, Gastanadui et al. [133] showed that plaque instability is closely related to the phenotypic transformation of SMCs and macrophage transdifferentiation. The schematic diagram of these studies is shown in Fig. 2A.

### Applications in myocardial infarction

Myocardial infarction (MI) occurs when a portion of the heart muscle (myocardium) experiences tissue death (infarction) caused by an insufficient supply of oxygen to the myocardial tissues or ischemia [134]. In an early investigation, Lacraz et al. [135] used Tomo-seq to obtain high spatial resolution genomic expression features of the infarct zone, identifying new regulators of cardiac remodeling. They highlighted SOX9 as a key regulator of cardiac fibrosis and a potential therapeutic target for heart fibrosis. Kuppe et al. [136] have established a comprehensive multi-omics atlas of MI by integrating snRNA-seq, snATAC-seq, and spatial transcriptomics technologies. They identified 9 distinct cell clusters in the ischemic zones of MI and located these clusters within spatial niches, uncovering spatial dependencies between different cellular communities. Additionally, the study revealed interactions between macrophages and fibroblasts within the human heart. Building on the same dataset, Shen et al. [137] used spatial scRNA-seq to compare CMs from MI patients with those in control subjects, focusing on metabolic adaptations within surviving CMs in ischemic niches. They observed fewer surviving CMs in infarcted hearts, with down-regulation in energy and amino acid metabolism pathways, indicating unique metabolic responses to ischemic stress. Similarly, Amrute et al. [138] utilized multi-omics analyses to study human hearts with acute MI (AMI) and chronic heart failure (CHF). They identified a disease-associated fibroblast subtype with high *FAP* expression and demonstrated interactions between *CCR^2+^* macrophages and fibroblasts through interleukin-1β (IL-1β) signaling, promoting fibroblast differentiation into profibrotic cells [138]. Further study conducted by Patrick et al. [139] integrated scRNA-seq and spatial transcriptomics data from multiple fibrosis-related MI studies in mice and humans (including data from Kuppe et al. [136]) and discovered key fibroblast characteristics in the progression and reversal of cardiac fibrosis.

Immune cells represent another major focus in MI research. Jung et al. [140] utilized spatial transcriptomics to monitor the dynamic changes in immune cell populations during MI, highlighting the temporal variations in the proportions of immune cells and uncovering significant spatiotemporal heterogeneity in macrophages. They speculated on the role of macrophages in MI, particularly noting an increase in the anti-inflammatory *Trem2* receptor in later stages of the condition. Furthemore, in a research conducted by Yamada et al. [141], employing a mouse model of MI for spatial transcriptomics and snRNA-seq analysis, the elevated expression of the mechanosensing gene Csrp3 at the periphery of the infarcted areas was discovered, suggesting Csrp3 as a potential therapeutic target for preventing ventricular remodeling post-MI. Boileau et al. [142] innovatively employed single-cell nanopore spatial transcriptomics (SCNAST) to study post-MI in the mouse heart. It was revealed that there was a significant regional isoform switching among differentially used transcripts of genes involved in myocardial contraction and tissue morphogenesis. Xu et al. [143] utilized scRNA-seq and spatial transcriptomics to characterize the temporal and spatial changes in mouse cardiac macrophage subtypes in response to MI, revealing the crucial role of BHLHE41^+^ macrophages in inhibiting myofibroblast activation, preventing excessive fibrosis, and limiting infarct expansion. Ninh et al. [144] analyzed MI models in mice and human cardiac tissue samples using Visium and MERFISH, revealing that CMs trigger the formation of interferon-induced cell clusters in the border zone through IRF3 activation, which disrupts post-infarction repair processes. Li et al. [145] studied CMs expressing active YAP in mouse hearts. Through spatial transcriptomics and scRNA-seq, a conserved, regenerative CM population colocalized spatially with cardiac fibroblasts expressing the C3 and macrophages expressing *C3AR1* was discovered, forming a niche that promotes myocardial regeneration. Wang et al. [146] discovered that gelsolin is a critical regulator of ventricular remodeling after MI by utilizing CRISPR/Cas9 technology and spatial transcriptomics in mice. The forementioned research represents a crucial step forward in understanding the cellular and molecular complexity of MI, potentially guiding the development of targeted therapeutic strategies. The schematic diagram of these studies is shown in Fig. 2B.

### Applications in cardiomyopathy

In the latest edition of the 2023 ESC Guidelines for the Management of Cardiomyopathies released by the European Society of Cardiology, cardiomyopathies are classified into 5 phenotypes: hypertrophic cardiomyopathy (HCM), dilated cardiomyopathy (DCM), nondilated left ventricular cardiomyopathy (NDLVC), arrhythmogenic right ventricular cardiomyopathy (ARVC), and restrictive cardiomyopathy (RCM) [147].

Arrhythmogenic cardiomyopathy, partially classified under ARVC, is a genetic heart disease characterized by myocardial loss, which is replaced by fibro-fatty cells, leading to arrhythmias and sudden cardiac death [148]. Boogerd et al. [149] delved into the specific remodeling processes associated with the development of ARVC, identifying new genes and pathways through the use of Tomo-seq to generate a transmural gene expression profile from the epicardium to the endocardium. They found that *ZBTB11* is specifically enriched at the sites of active fibro-fatty replacement in the myocardium, and that overexpression of *ZBTB11* can induce autophagy and cell death-related gene programs in human CMs, leading to increased apoptosis. This emphasizes the importance of understanding the local gene expression changes in ARVC.

HCM is the most common genetic heart disease, characterized by CM hypertrophy and cardiac fibrosis [150,151]. Liu et al. [152] performed snRNA-seq and spatial transcriptomics analysis on cardiac tissues from HCM patients, identifying the potential key genes (*FGF12*, *IL31RA*, and *CREB5*) involved in the transition process of CMs to a failure state, and the potential key genes (*AEBP1*, *RUNX1*, *MEOX1*, *LEF1*, and *NRXN3*) involved in cardiac fibrosis. They also confirmed that the spatial activity patterns of these genes and the NPPB^high^ CM subpopulation were near the fibrotic regions, revealing lineage-specific regulatory changes in HCM. Similarly, Laird et al. [153] analyzed the spatial transcriptomics of focal disordered regions of HCM tissue compared to normal tissue areas. The results showed significant changes in gene expression between HCM and control tissues. Compared to normal areas in HCM samples, significant differences in the composition of fibroblasts and vascular cells in severely disordered areas were revealed, revealing spatial heterogeneity and potential therapeutic targets in the disease progression of HCM. The schematic diagram of these studies is shown in Fig. 2C.

### Applications in heart failure

Heart failure (HF) is a syndrome caused by impaired cardiac blood pumping function. Many cardiovascular diseases can progress to HF, including coronary artery disease, MI, hypertension, atrial fibrillation, and valvular heart disease. HF can also be caused by excessive alcohol consumption, infections, and cardiomyopathies [154]. Asp et al. [155] have detected spatial variations between regions in the adult heart through spatial transcriptomics. They found spatial differences in the expression of fetal marker genes expressed by minor cell subpopulations within tissues in patients with HF with preserved ejection fraction. Additionally, Ko et al. [156], through the use of scRNA-seq and spatial transcriptomics analyses, elucidated the critical role of the HTRA3–TGF-β–IGFBP7 axis in regulating CM homeostasis, preventing cardiac fibrosis in MI areas, and inducing a secretory phenotype in CMs, demonstrating its potential as a therapeutic target for HF. Likewise, Linna-Kuosmanen et al. [157] employed snRNA-seq and spatial transcriptomics to study human right atrial tissues from patients with ischemic heart disease and HF, uncovering spatial features of atrial microvascular dysfunction and pro-inflammatory gene expression. Through spatial transcriptomics, researchers can identify region-specific gene expression signatures, which could be linked to the progression of HF and the development of targeted therapies. The schematic diagram of these studies is shown in Fig. 2D.

### Applications in cardiac myxomas

Cardiac myxomas are the most common primary cardiac tumors in adults, typically benign and attached to the left atrial septum, requiring prompt diagnosis and surgical removal due to potential life-threatening complications [158]. Liu et al. [159] dissected the tumor microenvironment in atrial myxomas through scRNA-seq and spatial transcriptomics, identifying myxoma tumor cells including EC-like tumor cells (ETCs) and mesenchymal stem cell-like tumor cells (MTCs), and finding that the former were differentiated from the latter. Additionally, they uncovered spatial characteristics of cell communication and clonal differentiation among myxoma tumor cells. The schematic diagram of the study of cardiac myxoma is shown in Fig. 2E.

### Applications in aortic dissection

Aortic dissection is a life-threatening condition caused by a tear in the intimal layer of the aorta or bleeding within the aortic wall, resulting in the separation of the layers of the aortic wall, characterized by acute onset, rapid progression, high mortality, poor prognosis, and increasing incidence rates [160]. Our team studied the gene expression characteristics from the tear area to the remote area of thoracic aortic dissection (TAD) using Tomo-seq, discovering significant spatial separation in gene expression across the lesion range [161]. Genes involved in immune processes were up-regulated in the tear area, while genes that are up-regulated in the remote area were primarily involved in repair functions. Furthermore, the study found that NINJ1 may be involved in inflammation and tissue remodeling, playing a important role in the formation of TAD. The schematic diagram of the study of aortic dissection is shown in Fig. 2F.

### Applications in viral myocarditis

Myocarditis, also known as inflammatory cardiomyopathy, is an acquired cardiomyopathy resulting in an inflammation of the heart muscle caused mostly by a viral infection [162]. Margaroli et al. [163] utilized scRNA-seq and spatial transcriptomics to thoroughly describe the cardiac gene expression changes associated with COVID-19, comparing myocardial samples from COVID-19 patients to those from a healthy control group. This comparison revealed distinct expression patterns linked to the disease. The spatial resolution of gene expression profiles from the epicardium to the endocardium provided insights into regional responses and remodeling processes during infection. In research by Kulasinghe et al. [164], spatial transcriptomics was used to analyze cardiac tissues from COVID-19 patients. Despite not detecting SARS-CoV-2, the up-regulation of genes related to DNA damage and repair, heat shock, and M1-like macrophage infiltration in the cardiac tissues of the patient sample group was found. This highlights the pro-inflammatory effects of the coronavirus on the cardiovascular system of patients and its long-term health impacts. Spatial transcriptomics, therefore, has emerged as a crucial tool in cardiovascular research, offering a unique perspective on the molecular and cellular consequences of diseases like COVID-19.

### Applications in congenital heart disease

Congenital heart defects (CHDs) refer to the structural abnormalities in the heart or major blood vessels present at birth, and are the most common type of congenital defect [165]. The exploration of CHD has entered a new era with the advent of spatial transcriptomics. For example, Kathiriya et al. [166] discovered overly sensitive dysregulation of *TBX5*-dependent pathways in CM subpopulations, revealing through spatial transcriptomic maps the chamber-restricted expression of *TBX5*-sensitive transcripts. This indicates a fine and diverse sensitivity to TBX5 dosage. Based on these findings, the researchers predicted a candidate gene regulatory network for human CHD. Existing research has also shown that homozygous missense mutations in the mouse low-density *LRP1* can lead to CHD [167]. Arrigo et al. [168], through a comprehensive analysis of public scRNA-seq datasets and spatial transcriptomics of human and mouse hearts, found that *LRP1* is primarily expressed in mesenchymal cells and is predominantly located in the developing outflow tract and atrioventricular cushions. Spatial transcriptomics underscores the importance of spatial context in gene expression, which is paramount for deciphering the pathophysiological mechanisms underlying CHD.

### Applications in other cardiovascular diseases

In addition to the cardiovascular diseases mentioned above, spatial transcriptomics has also been applied in the research of vasculitis, cardiac sarcoidosis, aortic coarctation, pulmonary arterial hypertension, and aortic aneurysms, among other conditions. Vasculitis is a group of disorders that destroy blood vessels through inflammation [169]. Both arteries and veins are affected. Porritt et al. [170] employed scRNA-seq in conjunction with spatial transcriptomics analysis to intricately display the visualization of various cellular populations within vascular tissues in a model induced with lipopolysaccharide to mimic conditions similar to vasculitis. Spatial maps showed the activation of the *NLRP3* inflammatory pathway and revealed the expression characteristics of key cell types in vasculitis, such as monocytes/macrophages/dendritic-like cells, 2 types of SMCs, and fibroblasts. The integration of spatial transcriptomics into vasculitis research provides a more comprehensive understanding of the pathology and paves the way for targeted therapeutic strategies that could mitigate the inflammatory response in vasculitis.

Sarcoidosis is a disease in which inflammatory cells abnormally aggregate to form masses called granulomas [171]. The disease usually begins in the lungs, skin, or lymph nodes, but the heart can also be affected. Liu et al. [172] utilized spatial transcriptomics and snRNA-seq to elucidate the cellular and transcriptional landscapes of cardiac sarcoidosis, comparing the transcriptomic maps of CD68^+^ immune cell infiltrates in human cardiac sarcoidosis, giant cell myocarditis, and lymphocytic myocarditis, and determining their spatial relationships. This research highlights the intricate relationships between macrophage phenotype, activation state, and spatial localization, providing insights into the dynamic cellular interactions during vascular inflammation.

Coarctation of the aorta is a congenital condition, a narrowing of the aorta, usually in the area where the arterial catheter is inserted, most commonly in the aortic arch [173]. Spatial transcriptomics has revolutionized our understanding of the vascular microenvironment. Li et al. [174] use spatial transcriptomics to dissect the heterogeneity and spatial organization of cellular populations within the arterial wall. This study identifies the distribution and gene expression profiles of various cell types including fibroblasts, SMCs, endothelial cells (ECs), and macrophages in the tunica intima, tunica media, and tunica adventitia. Moreover, the study further demonstrates the capability of spatial transcriptomics providing a visualization of the exact locations of these cells within the arterial architecture and insights into their functional roles and interactions in health and disease.

Liu et al. [175], through scRNA-seq and spatial transcriptomics analysis, discovered the transformation from capillary endothelium to arterial endothelium in patients with pulmonary arterial hypertension and animal models, located the spatial changes of ECs, and indicated that targeting arterial EC might be a new option for the treatment of pulmonary arterial hypertension. Mizrak et al. [176] analyzed the single-molecule spatial distribution of 140 gene transcripts in human thoracic aortic aneurysm (TAA) samples. They identified a characteristic spatial enrichment of SMC subpopulations expressing *CARTPT* in male TAA samples and further demonstrated that human *CART* promotes the osteochondrogenic switch in aortic SMCs, potentially leading to medial calcification in the thoracic aorta. Liu et al. [177] utilized snRNA-seq, snATAC-seq, and spatial transcriptomics to uncover the phenotypic regulatory dynamics of vascular smooth muscle cells (VSMCs) in aortic root aneurysms of Marfan syndrome (MFS) patients, identifying *FOXN3* and other regulatory factors as critical for maintaining the contractile phenotype of VSMCs. Vyas et al. [178] integrated CITE-seq (cellular indexing of transcriptomes and epitopes by sequencing), single-cell T cell receptor sequencing (scTCR-seq), and GeoMx to identify 2 resident memory T cell subpopulations in the epicardial adipose tissue of atrial fibrillation patients that promote atrial fibrillation susceptibility, and revealed their regulatory roles in myocardial calcium signaling and inflammatory pathways.

### Spatial transcriptomics in surgical transplantation research

Michaud et al. [179] utilized snRNA-seq and spatial transcriptomics to analyze the genomic effects of harvesting, distention, and implantation in the venous regions of vein grafts, revealing distinct regulatory programs and cell subpopulations mediating the response to acute distension injury. They discovered that distention initiated the up-regulation of pathological pathways, leading to bypass graft failure. Nevarez-Mejia et al. [180] conducted spatial transcriptomics and proteomic analyses on arterial regions of allogeneic heart transplants (AHTs) with donor-specific antibodies (DSAs) positive for cardiac allograft vasculopathy (CAV). They identified specific pathways and transcripts with enhanced inflammatory features within CAV lesions and pinpointed proteomic and transcriptomic characteristics distinguishing CAV lesions, further uncovering the immune mechanisms mediating the pathogenesis of CAV. Wang et al. [181] discovered that *FHL1* regulates vein graft neointimal hyperplasia by utilizing spatial transcriptomics and genetic manipulation in mice models and human saphenous veins.

### Spatial transcriptomics in drug-related research

Lin et al. [182] established a mouse model of myocardial ischemia–reperfusion (I/R) injury (IRI) and conducted snRNA-seq and spatial transcriptomics studies on mouse cardiac tissue to assess the effects of the traditional Chinese medicine She Xiang Bao Xin Pill (SBP) on infarct zone cell populations. They discovered that SBP was able to increase the expression of the NPPB gene within the cardiac infarct area and increase the number of fibroblasts as well as the conversion of ECs to fibroblasts.

## Spatial Proteomics in Cardiovascular Research

The application of spatial proteomics in cardiovascular diseases is limited and mainly used for exploring the spatial distribution of proteins and their link to functional roles and disease progression. A summary of spatial proteomics studies in cardiovascular disease is shown in Table 4.

**Table 4.** Summary of spatial proteomics in the research of cardiovascular diseases

Disease	Species	Year	Platform	Key target	Primary discoveries	Refs.
Studies using human samples						
Aortic stenosis	Human	2016	Unbiased MS-based spatial proteomics	Aortic valve tissue	Distinct structures corresponding to regions observed in conventional histology, such as large calcification areas and zones abundant in collagen and elastic fibers.	[183]
CAVD	Human	2018	Unbiased MS-based spatial proteomics	Aortic valve microlayers	The fibrotic stage-specific proteome identified pathways involves VIC myofibrogenesis and oxidative stress in early/fibrotic CAVD.	[184]
Connective tissue disorders	Human	2021	Unbiased MS-based spatial proteomics	Cardiac valves	The m/z values of aortic alpha smooth muscle actin and myosin heavy chains significantly increase in bicuspid aortic valve compared to MFS and tricuspid aortic valve	[185]
Viral myocarditis	Human	2022	Unbiased MS-based spatial proteomics	Inflammatory myocardia and microvessels	DISCO-MS enables comprehensive 3D proteome analysis of whole specimens for disease diagnosis left atrial myocardia and microvessels are most vulnerable to SARS-CoV-2 inflammatory injury.	[187]
Acute arterial thrombus	Human	2023	Unbiased MS-based spatial proteomics	Stiff and soft regions of arterial thrombus	Inhibition of TGF-β1 resulted in delayed arterial thrombosis and accelerated blood flow restoration.	[188]
MI	Human	2024	SeqIF, DVP	Endocardial regions	Immune cells infiltrate the infarcted heart through the endocardium via vWF-mediated adhesion, providing a novel target for post-infarction therapy	[192]
Atherosclerosis	Human	2024	Akoya PCF	SMCs	Smooth muscle cell-derived foam cells dominate early atherosclerosis and are linked to inflammatory processes within arterial walls	[195]

Studies using samples from other species						
MI	Murine	2022	Unbiased MS-based spatial proteomics	Infarct tissue of the core and border regions	The infarct core showed a significant down-regulation of cardiac biomarkers and an up-regulation of coagulation and immune response proteins compared to unaffected tissue.	[190]
MI	Murine	2024	AutoSTOMP	Fibroblasts, marcophages	Dynamic changes in whole-infarct proteome were captured, revealing that some protein composition signatures were differentially localized near SMA^+^ fibroblasts or CD68^+^ macrophages within the scar region.	[191]
Cardiac IRI	Murine	2023	Unbiased MS-based spatial proteomics	ECs	Increased H3K9me3 is identified as a key regulatory response in ECs during the middle stage of IRI.	[194]

In current spatial proteomics research on the cardiovascular system, unbiased mass spectrometry-based spatial proteomics predominates. Mourino-Alvarez et al. [183] directly obtained the spatial distribution of proteins and peptides from tissue in situ in consecutive sections of narrowed aortic valve tissues through spatial proteomics. They discovered a large number of calcified areas and areas rich in collagen and elastin fibers enriched with collagen VIα-3 and NDRG2 proteins, proving that spatial proteomics can provide spatial resolution in situ. Schlotter et al. [184] classified samples from 25 human calcific aortic valve disease (CAVD) into time-specific samples of disease progression stages using spatiotemporal multi-omics (including proteomics and transcriptomics) and captured spatially specific information through LCM, characterizing the spatiotemporal differences in the CAVD progression process. They established protein interaction and molecular regulatory networks, identifying central proteins and disease associations, providing a research strategy for multi-omics studies of cardiovascular diseases. Mohamed et al. [185] characterized the spatial changes in the ascending aorta tissue through spatial proteomics based on MALDI-MS, finding distribution differences in characteristic peptides between different diseased valve elastic fiber fragmentation, confirmed by IHC. Bhatia et al. [186] introduced the DISCO-MS technique, which allows for unbiased proteomic analysis of preclinical and clinical tissues after 3D panoramic image acquisition. They applied this technique to study aortic plaques in human hearts, offering opportunities for complex disease diagnosis and treatment. Leng et al. [187] established myocardial and microvascular area-resolution proteomic maps of the myocarditic hearts of COVID-19 patients using spatial proteomics strategies, discovering inflammatory cells in the microvasculature. Additionally, molecular dysfunction in the myocardium and microvasculature was found in various heart regions, with the left atrial wall being most susceptible to viral infection-induced inflammation, a finding of therapeutic relevance. Mai et al. [188] combined proteomics with LCM to analyze human acute arterial thrombi to identify hard and soft areas. Through spatial proteomic analysis, they proved that the protein composition of hard and soft areas in human arterial thrombi is spatially distinct, identifying TGF-β1 as a key therapeutic target for promoting arterial thrombolysis. Reperfusion therapy is an effective way to restore blood supply and oxygen to ischemic tissues, but IRI can exacerbate myocardial damage, and the pathological mechanisms of myocardial IRI are not yet clear. Currie et al. [189] measured protein turnover rates and subcellular localization simultaneously through a mass spectrometry-based spatial proteomics method, applied to an induced pluripotent stem cell-derived CM (iPSC-CM) model treated with the proteasome inhibitor carfilzomib in addition to cardiotoxic anticancer drugs. They found that carfilzomib did not reduce protein half-lives but effectively disrupted sarcomere protein homeostasis. This study provides new insights into the application of spatial proteomics technology in disease model research using iPSC-CM.

Several studies have utilized spatial proteomics to investigate MI. Mezger et al. [190] revealed murine ischemia-specific regions through spatial proteomics, displaying distinct contours of the infarction core and border. They discovered significant down-regulation of myocardial biomarkers and up-regulation of coagulation and immune response proteins in the infarction core. This research, through the mining spatial information in tissue sections for protein analysis, proposes new ideas for exploring the mechanisms of myocardial IRI and mitigating post-MI damage. Mallikarjun et al. [191] used the automated spatially targeted optical micro-proteomics (autoSTOMP) technique for targeted labeling of fibroblast and macrophage-associated proteins in murine MI tissue. By tracking the progression of MI, they explored the dynamic changes in the infarct proteome, finding its protein composition characteristics related to the different localizations of SMA^+^ fibroblasts or macrophages in the scar area, further illustrating the potential of spatial proteomics in exploring the mechanisms of progression in cardiovascular diseases. Wünnemann et al. [192] isolated cells from the endocardial regions of healthy and post-infarction hearts in humans and mice using LCM, followed by spatial proteomics analysis with sequential immunofluorescence (SeqIF) and deep visual proteomics (DVP) platforms, identifying and quantifying cell type distributions, differentially expressed proteins, and their potential functions.

ECs, ubiquitous in the cardiovascular system, have been found through scRNA-seq to include shear stress-related ECs, indicating the notable impact of shear stress on the physiological functions of vascular endothelium. Sewduth et al. [193] analyzed the spatial characteristics of the endothelial proteome through spatial proteomics to reveal the mechanisms of endothelial activation by shear stress. They found that the nondegradative ubiquitination of multiple guanosine triphosphatases (GTPases) is regulated by mechanical signals, demonstrating the importance of spatial regulation of nondegradative GTPase ubiquitination in endothelial activation pathways under shear stress. Yao et al. [194] mapped the cellular structure of mouse hearts after I/R at single-cell resolution and revealed the spatiotemporal regulatory map at the translational and posttranslational levels during myocardial I/R using spatial proteomics based on multichannel antibody staining imaging mass spectrometry technology. They found that H3K9me3 in ECs is an important potential target for treating pathological remodeling after I/R.

Antibody-based targeted spatial proteomics methods are currently less frequently applied. In a recent study, Elishaev et al. [195], on the other hand, used the Akoya PCF spatial proteomics platform to quantitatively analyze foam cell types and their spatial distribution in early human atherosclerotic lesions, finding that IL-1β^high^ SMCs exhibit pronounced inflammatory characteristics and are associated with foam cell formation and apoptosis.

The field of spatial proteomics is gradually unraveling the intricate molecular landscapes within cardiovascular diseases, providing profound insights into disease mechanisms, progression, and potential therapeutic targets. From uncovering specific protein distributions in heart diseases to identifying key molecular networks in disease states, spatial proteomics is poised to revolutionize our understanding of cardiovascular pathology. However, challenges such as improving throughput and achieving subcellular resolution remain. Future advancements in spatial proteomics technologies and methodologies promise to enhance our ability to decipher the complex spatial and molecular dynamics of cardiovascular diseases, offering new avenues for diagnosis, treatment, and personalized medicine in cardiovascular diseases.

## Spatial Metabolomics in Cardiovascular Research

Metabolomics has been widely applied in the cardiovascular field. Researchers have mapped the lipid profiles of whole hearts at different developmental stages in mice, but there is a lack of detailed characterization of spatial information [196]. For spatial metabolomics, there are numerous reports on the study of atherosclerosis in the cardiovascular field. The heterogeneity of macrophages in atherosclerosis has been widely verified in related studies using scRNA-seq [197–200]. Guo et al. [201] discovered that rationally designed micelles significantly increase the accumulation of berberine (BBR) in the liver and demonstrated in mice on a high-fat diet that BBR-CTA-Mic not only effectively promotes liver cell uptake of BBR but also reduces plasma levels of pro-inflammatory cytokines and lipid content, thereby achieving a potential therapeutic effect on atherosclerosis. Goossens et al. [202], through spatial multiplex immunofluorescence imaging and spatial metabolomics techniques, divided the complex tissue cells in murine atherosclerotic plaques into different ecological niches based on local lipid metabolic characteristics, further revealing the heterogeneity of macrophages in atherosclerotic plaques through spatial metabolic differences. Seeley et al. [203] described the spatial distribution of metabolites in human stable and unstable atherosclerosis plaques. By integrating MSI with RNA sequencing data, they found that in stable plaques lipid metabolism and long-chain fatty acid metabolism pathways were enriched, with increased levels of acetylcarnitine and acetylglycine, increased lactic acid in the organic core area, and elevated pyruvate in the fibrous cap area. Furthermore, in unstable plaques, an increase in reactive oxygen species, aromatic amino acids, and tryptophan metabolism, enrichment of tryptophan metabolism products, and 5-hydroxyindoleacetic acid enriched in the fibrous cap area were found, providing important value for depicting the metabolic map of atherosclerosis and opening new avenues for cardiovascular disease research. Li et al. [204] used DESI-MSI to reveal the spatial characteristics of lipid metabolism in human carotid atherosclerotic plaques and their association with disease progression. Slijkhuis et al. [205], through spatial lipidomics, studied the lipid characteristics of human carotid artery plaques and plasma, mapping the spatial distribution of lipid-related m/z features within plaques and matching spatial lipid patterns with corresponding histological regions. This revealed specific lipids present in pig unstable plaque areas. In another study, they investigated the colocalization between lipids and histological regions in pig advanced disease artery segments and depicted the spatial distribution of lipids, uncovering lipid features associated with plaque development and colocalization with the necrotic core and inflammatory cells [206], providing more insight into the progression of coronary atherosclerotic disease. Cao et al. [207] studied the metabolic changes in the murine aortic plaques, media, arteries, and heart tissues of atherosclerotic mice from a spatial perspective, finding that atherosclerosis leads to changes in phospholipid metabolism, affecting the aorta and adjacent cardiac tissues. Through spatial metabolomics identification of tissues, they found that plaque progression-related lipids lysophosphatidylcholine (LPC) (18:0) and lysophosphatidic acid (LPA) (18:1) could reflect cardiovascular risk in human plasma.

Beyond atherosclerosis, spatial metabolomics is also applied in other cardiovascular diseases. Arterial restenosis is a common recurrence disease following cardiovascular interventions such as balloon angioplasty and stent implantation. Shi et al. [208] applied MALDI-MSI for spatiotemporal lipidomics studies in animal models of arterial restenosis, discovering significant increases in diacylglycerols and lysophosphatidylcholines in the neointima layer of arteries damaged by balloon injury. This provides new insights into the mechanisms of restenosis involving lipids or small signaling molecules.

Research has also been conducted on ischemia, which causes widespread lesions in the cardiovascular system. Wang et al. [209] conducted a spatial analysis of metabolic dysfunction in ischemic tissues and proposed a spatial metabolomics method of in situ ratio MSI, which can delineate the injury boundary and annotate the metabolic and enzymatic features in ischemic tissues. Through related experiments, this method has been proven to have high precision and robustness in identifying lesions in a murine cardiac ischemia model, demonstrating the potential for exploring ischemic damage in the cardiovascular system at the metabolic and enzymatic levels. Metabolic remodeling plays an important role in the progression of various cardiovascular diseases to HF. Ren et al. [210], using cardiac myocytes from transgenic HF mice for cell metabolic analysis, identified specific metabolic markers of HF through lipid landscape analysis, displaying their spatial distribution, further discovering their association with lipoprotein metabolism, transmembrane transport, and signaling. This illustrates that spatial metabolomics aids in identifying markers associated with cardiovascular diseases and provides more insight into related metabolic pathways. Diabetic cardiomyopathy is a metabolic disease leading to HF progression in the diabetic population. Liu et al. [211] visualized metabolites in rat hearts with high spatial resolution and sensitivity through spatial metabolomics technology, revealing metabolic heterogeneity in the heart under the diabetic cardiomyopathy model. This research provided a theoretical basis for potential drug treatment and development for diabetic cardiomyopathy.

As spatial omics technologies continue to advance, spatial metabolomics is anticipated to show potential application in the cardiovascular field by mapping the metabolic activities within the cardiovascular system with high precision. This will provide an in-depth understanding of cell interactions and metabolic pathways in the disease microenvironment and reveal tissue- and cellular-level metabolic changes, thereby aiding in the identification of early biomarkers and potential therapeutic targets for diseases. In addition, spatial metabolomics has great potential in drug development and therapeutic applications for cardiovascular diseases. For example, Cheng et al. [212] applied DESI-MSI technology to demonstrate that Chuanxiong promotes angiogenesis via the phosphatidylinositol 3-kinase (PI3K)/AKT/Ras/mitogen-activated protein kinase (MAPK) signaling pathways. It will play a crucial role in early diagnosis, unraveling disease mechanisms, and the development of new therapies and promote the development of personalized medicine by analyzing patient-specific metabolic features to tailor treatment plans, further enhancing treatment efficiency and safety. In summary, spatial metabolomics is poised to open a new chapter in the cardiovascular field. A summary of spatial metabolomics studies in cardiovascular disease is shown in Table 5.

**Table 5.** Summary of spatial metabolomics in the research of cardiovascular diseases

Disease	Species	Year	Platform	Key target	Primary discoveries	Refs.
Studies using human samples						
Atherosclerosis	Human	2023	MALDI-MSI	Atherosclerotic plaques	Defining an atlas of metabolic pathways involved in plaque destabilization in human atherosclerosis.	[203]
Atherosclerosis	Human	2023	DESI-MSI	Lipid	DESI-MSI unveils distinct lipid distributions and metabolic pathways in carotid atherosclerotic plaques, correlating with pathological progression	[204]
Atherosclerosis	Human	2023	DESI-MSI	Atherosclerotic plaques	Notable lipid species in plasma were found in carotid plaque regions linked to plaque destabilization.	[205]
Atherosclerosis	Human	2024	MALDI-MSI	Lipid	LPC(18:0) and LPA(18:1) reflect plaque-progression and cardiovascular risk in human plasma.	[207]
Studies using samples from other species						
Atherosclerosis	Murine	2019	MALDI-MSI	BBR	BBR-CTA-Mic intervention remarkably improves metabolic profiles and reduces the formation of aortic arch plaque.	[201]
Atherosclerosis	Murine	2022	MALDI-MSI	Macrophages	Revealing murine atherosclerotic plaque myeloid heterogeneity and allowing further histological characterization with cellular and molecular microenvironmental contexts.	[202]
Atherosclerosis	Pig	2024	MALDI-MSI	Atherosclerotic plaques	Uncovering distinct lipid signatures correlated with plaque development and their colocalization with necrotic core and inflammatory cells.	[206]
Arterial restenosis	Murine	2019	MALDI-MSI	Neointimal layer of balloon-injured arteries	Revealing distinct temporal-spatial dynamics of lipids functionally related to restenosis.	[208]
Ischemia	Murine	2021	AFADESI-MSI	Ischemic tissue	A ratiometric MSI method has been established for convenient in situ metabolomics analysis of tissue ischemia and highly efficient screening of enzymatic alterations in energy metabolism.	[209]
HF	Murine	2023	ToF-SIMS	CMs	A series of metabolic features were identified and used for discrimination of the HF CMs.	[210]
Diabetic cardiomyopathy	Murine	2023	AFADESI-MS; MALDI-MSI	Metabolic profile	Uncovering the heterogeneous metabolic profile of the heart in the Diabetic cardiomyopathy model, with over 105 region-specific alterations in various metabolite classes.	[211]

## Conclusion and Future Prospects

**Fig. 3.**
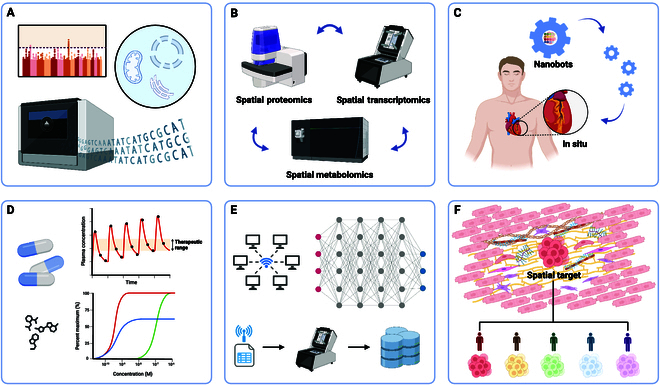
Future prospects of spatial omics technologies. (A) Enhanced sequencing depth and detection throughput. Advancements in sequencing technology will enable higher spatial resolution and detection throughput at single-cell or subcellular levels. (B) Integration of spatial multi-omics. Platforms like SpatialGlue, SLAT, and GENIUS integrate spatial multi-omics data, enhancing accuracy and scalability in identifying spatial domains and cell types. (C) In situ spatial multi-omics detection. In situ spatial multi-omics detection will address sample quality loss, with nanorobots offering potential for in vivo research. (D) Drug research applications. Spatial multi-omics will elucidate drug pharmacokinetics, pharmacodynamics, and toxicological effects, aiding drug research and development. (E) Automation and AI. AI advancements will automate complex spatial omics processes, reducing costs and improving efficiency. (F) Personalized precision therapy. Spatial multi-omics may identify “spatial target groups” for personalized precision therapy, guiding individualized treatment. Created with http://BioRender.com.

Spatial omics has progressively evolved into a crucial tool for research on the cardiovascular system and cardiovascular diseases, uncovering the spatial and molecular heterogeneity that underpins cardiac function and pathology. Spatial omics remains an emerging technology with tremendous development prospects. The future prospects of spatial omics technologies are illustrated in Fig. 3. Future advancements in sequencing technology will likely improve spatial resolution, sequencing depth, and detection throughput at both single-cell or subcellular levels (Fig. 3A). Currently, there are studies combining spatial omics with other multi-omics technologies [3]. These technologies are extensively utilized in oncology research, including studies on gastric cancer [213], lung cancer [214], and breast cancer [215]. In the cardiovascular field, some studies have integrated spatial omics with single-cell assay for transposase-accessible chromatin (sc-ATAC) data [112,136]. However, research that combines multiple spatial omics datasets remains relatively limited. With the introduction of more multiomics integration platforms, it is reasonable to believe that spatial multiomics will be more widely applied in the future (Fig. 3B). In addition, spatial multiomics detection in tissues or even in vivo can directly address sample losses during acquisition and preparation. Currently, the emergence of nanorobots offers new possibilities for automation in in vivo or in situ spatial multiomics research (Fig. 3C). Drug research can also benefit from spatial multiomics technology, revealing the pharmacokinetics, pharmacodynamics, and toxicological effects of drugs (Fig. 3D). AI has numerous applications in the biomedical field, including deep learning-based microfluidic organs-on-chips (OoCs) [216], deep graph learning models for identifying ligandable covalent sites [217], drug research at the single-cell level [218], protein function prediction [219] and structural analysis [220], as well as direct analysis via DIA [221]. With the development of AI, the complex steps of spatial omics technology are expected to become automated in the future, reducing costs and improving efficiency (Fig. 3E). Spatial multiomics has been widely used to explore the microenvironment, especially in the field of tumor microenvironments [222]. In the cardiovascular field, Kanemaru et al. [112] discovered unique microecological structures in the sinoatrial node and the marked “signal hub” role of macrophages in the human epicardial immune microecology. As the importance of precision medicine and prevention is increasingly recognized, identifying individualized therapeutic targets will be a primary focus in future clinical practice, where single-cell omics and spatial omics provide great potential. We propose that spatial multiomics may discover spatially characteristic individualized “spatial targets” in the future (Fig. 3F). The novel concept of “spatial targets” is defined as niches of therapeutically relevant molecules, metabolites, or cells organized spatially.

Looking ahead, several promising research areas and key technical breakthroughs are poised to shape the next phase of spatial omics in cardiovascular studies. First, improvements in labeling and detection methods—such as molecular barcoding and in situ sequencing—will further enhance single-cell or subcellular resolution and allow more precise temporal measurements [223]. Second, developing robust computational frameworks and AI-driven algorithms will be critical for integrating large-scale, multimodal datasets while minimizing artifacts and data loss [107]. These advanced computational tools will facilitate the analysis of complex 3D and 4D datasets, enabling more accurate modeling of cellular interactions and temporal dynamics within the cardiovascular system, particularly in the areas of cardiovascular development, aging, and the progression of cardiovascular diseases. Additionally, microfluidic-based automation combined with micro-sampling techniques may streamline both ex vivo and in vivo spatial multiomics applications [224]. For example, emerging technologies like expansion microscopy (ExM), which physically expands biological samples for ultra-high-resolution 3D imaging, will reduce experimental complexity and increase stability, allowing for high-throughput and reproducible analyses of cardiovascular tissues in 3 dimensions and over time [225]. The convergence of these technologies will accelerate discoveries in cardiovascular biology and disease, paving the way for novel diagnostic and therapeutic strategies. We believe that identifying individualized spatial target groups can further guide personalized precision therapy in the future.
